# Spatial distribution of isoprenoid enzymes and MpABCG1 transporter influences sesquiterpene accumulation in *Marchantia polymorpha* oil bodies

**DOI:** 10.1038/s42003-025-09508-4

**Published:** 2026-03-02

**Authors:** Edith C. F. Forestier, Paola Asprilla, Ignacy Bonter, Facundo Romani, Eftychios Frangedakis, Jim Haseloff

**Affiliations:** Department of Plant Sciences, University of Cambridge, Cambridge, United Kingdom

**Keywords:** Secondary metabolism, Plant molecular biology

## Abstract

*Marchantia polymorpha* oil bodies (OBs) are specialized cell structures housing a diverse array of C15-terpenes, called sesquiterpenes. These compounds act as herbivore repellents, yet the enzymes responsible for the biosynthesis of their precursors remain poorly characterized. We investigated the localization of isoprenoid biosynthetic enzymes using translational and transcriptional reporters, coupled with confocal microscopy. Most enzymes localized as predicted (*e.g*., cytosol, plastid and the endoplasmic reticulum), and OB cells were identified as the primary sites of sesquiterpene biosynthesis. To explore OBs as potential storage sites for terpenes, we attempted to produce exogenous but easily identifiable compounds in *Marchantia*, such as the diterpene taxadiene and the triterpene β-amyrin. Targeting to OB cells resulted in measurable amounts of these compounds, but their yields remained unaffected by the overexpression of key precursor genes, underscoring challenges in redirecting metabolic flux. To further investigate terpene accumulation in OBs, we focused on MpABCG1, an ABC transporter previously reported to localize at the OB membrane. While Mp*ABCG1* overexpression mildly increased endogenous sesquiterpene levels, its disruption via CRISPR dramatically reduced sesquiterpene accumulation. These findings establish that MpABCG1 is necessary for sesquiterpene accumulation in OBs and add to current knowledge of terpene synthesis compartmentalisation in *Marchantia polymorpha*.

## Abstract (web summary)

A study maps terpene biosynthesis in *Marchantia polymorpha* oil bodies and identifies the ABC transporter MpABCG1 as a key factor for endogenous sesquiterpene accumulation in oil bodies, informing future metabolic engineering strategies.

## Introduction

In recent years, *Marchantia polymorpha* has emerged as a useful model plant system^1^ for studying terpene biosynthesis and accumulation within specialized storage structures known as oil bodies (OBs)^2^. The OBs in *Marchantia* are membrane-bound compartments that primarily house terpenes^3^ and bisbibenzyls^4^, a class of phenylpropanoid derivatives; the mixture confers anti-feedant properties to protect against herbivores^5^. Terpenes are a diverse class of natural products derived from the condensation of isoprene (C_5_H_8_) units^6^, found across all life, with the greatest structural and functional diversity observed in the plant kingdom. Beyond deterring herbivores as observed in *Marchantia*, they serve other vital roles in plants, such as preventing fungal infections or attracting pollinators. Additionally, a broad range of bioactive properties makes them valuable in medicine, agriculture, food and other industrial applications.

The sequestration of bisbibenzyls and terpenes into OBs is critical for protecting *Marchantia* from the potential toxicity of these metabolites when released into other tissues. The unique compartmentalization offered by OBs presents a promising avenue for metabolic engineering, as it provides a natural reservoir for accumulating metabolites that might otherwise be harmful to the plant.

Recent developmental studies have provided significant insights into the mechanisms governing OB cell fate determination and formation, highlighting key transcription factors (TFs) involved in these processes. Specific TFs, such as MpERF13^7^, MpC1HDZ^5^, MpTGA^8^ and MpMYB2^9,10^ have been identified as crucial regulators of OB cell differentiation and maturation^11^. These discoveries have expanded our understanding of how OB cells develop and contribute to the plant's metabolic capacity.

Manipulating these TFs has further revealed the delicate balance between OB cell proliferation and plant fitness. For instance, CRISPR-mediated mutation of Mp*TGA*^8^ or gain-of-function mutants of Mp*ERF13* led to a dramatic increase in OB cell numbers per plant^7^. However, these alterations were accompanied by reduced growth rates or morphological defects, suggesting that OB cell number is tightly regulated in *Marchantia* to avoid compromising overall plant health and fitness, or that these TFs have pleiotropic effects that influence additional developmental processes.

Early studies on *Marchantia’s* terpene biosynthetic pathway, which were based on immunolocalization of isoprenoid enzymes, hypothesized that key enzymatic steps might occur at the oil body (OB) membrane^3^. Today, with the availability of whole-genome sequencing and detailed gene annotations informed by sequence homology with other species, our understanding of the early steps of isoprenoid biosynthesis has significantly improved^12–14^ (Table 1 and S1). The mevalonate (MVA) and methylerythritol phosphate (MEP)^15–17^ pathways are the two primary metabolic routes responsible for producing the precursors—isopentenyl pyrophosphate (IPP) and dimethylallyl pyrophosphate—for various classes of terpenes (Table 1). The MVA pathway, comprising seven enzymatic steps^18^ and typically operating in the cytosol^19^, endoplasmic reticulum^20^ (ER) and peroxisome^21^, generally supplies precursors to sesquiterpenes and triterpenes synthesis^22,23^, while the MEP pathway, with seven/eight enzymatic steps and primarily localized in plastids^24^, commonly provides precursors for monoterpenes and diterpenes^22,23^ (Table 1 and S1). In *Marchantia*, sesquiterpenes are the dominant terpene class found in OBs^2^, produced by several microbial- and fungal-type terpene synthases^25^. Additionally, *Marchantia* produces the monoterpene limonene, likely synthesized through the cis-prenyl precursor neryl pyrophosphate rather than the transform geranyl pyrophosphate (GPP), as demonstrated by Kumar et al.^26^. While cell-type-specific metabolic analyses revealed that only sesquiterpenes are stored inside the OB compartment^2^, plants defective in OB cell differentiation show reduced levels of both sesquiterpenes and monoterpenes^5^, making it unclear whether limonene is specifically stored within OBs.

**Table 1:** Overview of MVA, MEP, and terpene scaffold biosynthetic genes in *Marchantia polymorpha*

	Name	Abbreviation	Putative localization (DeepLoc 2.1)
MEP	1-Deoxy-D-xylulose-5-phosphate synthase 1	Mp*DXS1*	Plastids/soluble
	1-Deoxy-D-xylulose-5-phosphate synthase 2	Mp*DXS2*	
	1-Deoxy-D-xylulose 5-phosphate reductoisomerase 1	Mp*DXR1*	
	1-Deoxy-D-xylulose 5-phosphate reductoisomerase 2	Mp*DXR2*	
	4-Diphosphocytidyl-2-*C*-methyl-*D*-erythritol synthase	Mp*MCT*	
	4-Diphosphocytidyl-2-*C*-methyl-*D*-erythritol 2-phosphate kinase	Mp*CMK*	
	2-*C*-methyl-*D*-erythritol 2,4-cyclodiphosphate synthase	Mp*MDS*	
	1-Hydroxy-2-methyl-2-*(E)*-butenyl 4-diphosphate synthase	Mp*HDS*	
	1-Hydroxy-2-methyl-2-*(E)*-butenyl 4-diphosphate reductase	Mp*HDR*	
	Isopentenyl diphosphate isomerase 1	Mp*IDI1*	
	Isoprenyl (geranylgeranyl) diphosphate synthase 2	Mp*IDS2*/Mp*GGPPS*	Plastids/soluble
	Isoprenyl diphosphate synthase 3	Mp*IDS3*	Plastids/soluble
	Isoprenyl (solanesyl) diphosphate synthase 4	Mp*IDS4*	Plastids/soluble
	Isoprenyl diphosphate synthase 5	Mp*IDS5*	Mitochondria/soluble
MVA	Acetoacetyl-coA thiolase 2	Mp*ACT2*	Peroxisome/soluble
	3-Hydroxy-3-methylglutaryl-coA synthase	Mp*HMGS*	Cytoplasm
	3-Hydroxy-3-methylglutaryl-coA reductase	Mp*HMG*/Mp*HMGR*	ER/transmembrane
	Mevalonate-5-kinase	Mp*MK*	Cytoplasm
	Phosphomevanolate kinase	Mp*PMK*	Cytoplasm
	Mevanolate diphosphate decarboxylase.	Mp*MVD*	Cytoplasm
	Isopentenyl diphosphate isomerase 2	Mp*IDI2*	Cytoplasm
	Farnesyl pyrophosphate synthase	Mp*IDS1*/Mp*FPS*	Mitochondria/soluble
	Squalene synthase 3	Mp*SQS3*	ER/transmembrane
	Squalene/phytoene synthase 1	Mp*SQS1*	Plastids/peripheral
	Squalene/phytoene synthase 2	Mp*SQS2*	Mitochondria/peripheral

This table lists key genes studied or referenced in this work, including those involved in the methylerythritol phosphate (MEP) and mevalonate (MVA) pathways, as well as enzymes responsible for synthesizing terpene scaffolds for different subclasses. Abbreviations correspond to gene names, and their putative protein localizations were predicted using DeepLoc 2.1. Detailed annotations and functional descriptions for each gene are provided in Table S1.

Examination of genome data^12–14,27^ along with computational predictive tools for enzyme localization, such as DeepLoc 2.1^28^, enabled us to identify putative precursor genes involved in prenyl phosphate synthesis for terpene production (Table 1). However, discrepancies remain between earlier theories suggesting that terpene biosynthetic enzymes are localized to OB membranes^3^ and more recent predictions that place MVA enzymes in the cytosol, ER and peroxisome, and MEP enzymes in plastids (Table 1). These discrepancies prompted us to re-examine the localization of key enzymes using modern techniques such as confocal microscopy combined with translational reporters^7^. This approach allowed us to identify the specific cells expressing these genes and determine the subcellular localization of their corresponding proteins.

Building on our findings regarding terpene precursor gene expression and localization, we then explored the potential for metabolic engineering in *Marchantia polymorpha*’s OBs. Specifically, we aimed to produce two valuable terpenes within OBs: the diterpene taxadiene^29^, a precursor to the valuable anti-cancer compound TaxolⓇ^30,31^, and the triterpene β-amyrin^32^, a precursor to compounds such as glycyrrhizin^33^, a sweet-tasting constituent of liquorice. These compounds were selected due to their distinct chemical signatures, which facilitate easy detection in chromatographic analyses, given the minimal background of these terpene subclasses in *Marchantia*. To further evaluate the metabolic capacity of *Marchantia* and investigate the transport of sesquiterpenes, we also examined the production of the sesquiterpene amorpha-4,11-diene^34^ alongside MpABCG1, an ATP-binding cassette (ABC) transporter highly and exclusively expressed in OB cells^35^. Prior studies have demonstrated its clear localization to the OB membrane using a translational reporter^7^, suggesting a potential role in metabolite transport. This study provides new insights into the involvement of MpABCG1 in terpene accumulation within OBs and contributes to a broader understanding of the partitioning of terpene metabolism in *Marchantia polymorpha*.

## Results

### Translational and transcriptional reporters of terpenoid precursor pathway genes

To determine the expression pattern and subcellular localization of key enzymes involved in *Marchantia*'s terpene biosynthesis, we generated constructs of translational reporters, in which each gene’s native promoter drove its respective coding sequence fused to the fluorescent tag *mVenus*^36^. In cases where translational reporters were unavailable due to technical challenges, transcriptional reporters were used instead to determine cell-type expression only. These consisted of *Marchantia*’s native promoter driving mVenus with a nuclear localization signal^37^ (mVenus-N7), enabling us to investigate the spatial and temporal expression of key genes. Promoter lengths were selected based on Assay for Transposase-Accessible Chromatin with Sequencing (ATAC-seq)^38^ data from version 6.1 of the Tak accession in the *Marchantia.info* database^13,25^ (Table S2). ATAC-seq identifies regions of open chromatin, which are more accessible to TFs and associated with regulatory domains that influence gene expression. Since relaxed chromatin is frequently found near the start of a gene, it serves as a useful indicator for selecting promoter regions likely to drive gene expression in their native cellular contexts. To delineate cell boundaries, each construct also included a fluorescent membrane marker, *mScarlet-LTI6b* fusion^39–41^, driven by the strong, constitutive *ubiquitin-conjugating enzyme E2* gene promoter and 5’UTR from *Marchantia polymorpha* (_*Pro*_*UBE2*)^41^.

For the identification of different subcellular compartments, such as chloroplasts/plastids, ER, cytosol, Golgi, and mitochondria, we interpreted our findings by comparing the localization patterns observed in our images to those of previously characterized standard reporters in *Marchantia*^41,42^, though distinguishing between the ER and cytosol remained challenging under the resolution constraints of our experimental setup.

For this study, we selected key genes from the MVA and MEP pathways on the basis of their predicted roles as rate-limiting enzymes, their redundancy, or their involvement in the final steps of the pathway. Additionally, we included genes responsible for the formation of linear scaffold precursors for sesquiterpenes, diterpenes, and triterpenes to capture a more comprehensive view of terpene biosynthesis. Among these, five genes were annotated as isoprenyl diphosphate synthases (*IDS*)^27^ (Table 1), which integrates functional annotation with phylogenetic relationships via its orthophyloviewer tool^27^. We used these data, alongside sequence comparisons with *Arabidopsis* homologs, to refine tentative annotations for *IDS* genes likely involved in terpene precursor formation for this study. The remaining genes analyzed here were already clearly annotated in Marchantia.info thanks to the extensive curation by Chen et al.^27^, and did not require further reannotation.

**Fig. 1:**
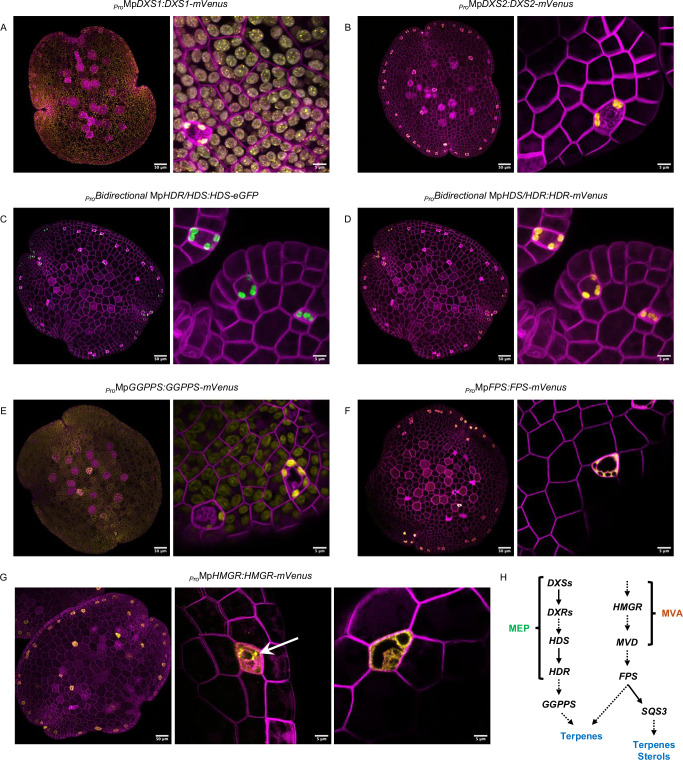
Confocal imaging of translational reporters for key *Marchantia polymorpha* isoprenoid biosynthetic genes. **A**_Pro_MpDXS1:DXS1-mVenus, **B**_Pro_MpDXS2:DXS2-mVenus, **C**_Pro_Bidirectional MpHDR/HDS:HDS-eGFP, **D**_Pro_Bidirectional MpHDS/HDR:HDR-mVenus, **E**_Pro_MpGGPPS:GGPPS-mVenus, **F**_Pro_MpFPS:FPS-mVenus, and **G**_Pro_MpHMGR:HMGR-mVenus. Each construct is represented by two images: the full gemmae at day 0 (left panels, scale bar: 50 μm) and a higher magnification view showing subcellular localization (right panels, scale bar: 5 μm). **H** Simplified biosynthetic pathway scheme highlighting enzymatic steps analyzed in this study. The mVenus signal (yellow) or eGFP (green) for MpHDS (**C**) indicates the subcellular localization of the corresponding enzymes, while mScarlet (purple) delineates the cellular boundaries. For the magnified image in (**A**), chloroplast/plastid autofluorescence (gray) is included to demonstrate sublocalization of MpDXS1 in the plastids. **G** includes one whole-gemma image and two high magnification images to illustrate the localization of MpHMGR-mVenus to the ER and Golgi apparatus. White arrow indicates the localizations to the Golgi apparatus.

We first analyzed the two annotated *1-deoxy-D-xylulose-5-phosphate synthases* (Mp*DXS1 and* Mp*DXS2*) (Table 1), which encode the first committed and rate-limiting enzymes of the MEP pathway^43^. For Mp*DXS1*, tagged with mVenus and driven by its native promoter and 5’UTR (_*Pro*_Mp*DXS1:DXS1-mVenus*), mVenus fluorescence was observed in all cell types throughout the plant, with a stronger signal in OB cells (Fig. 1A). The protein localized to plastids, displaying a punctate pattern in non-OB cells rather than a homogeneous distribution (Fig. 1A). By contrast, _*Pro*_Mp*DXS2:DXS2-mVenus* showed a restricted localization pattern, with fluorescence observed only in oil body plastids from Day 0 gemmae to 14-day-old plants (Figs. 1B and S1C).

We examined the final steps of the MEP pathway by generating a translational reporter for *(E)-4-hydroxy-3-methylbut-2-en-1-yl diphosphate reductase* (Mp*HDR*)^44,45^ (Table 1). We also generated a translational reporter for *(E)-4-hydroxy-3-methylbut-2-enyl diphosphate synthase* (Mp*HDS*)^44,46^ (Table 1). Mp*HDS* is adjacent to Mp*HDR* and oriented in the opposite direction on the same chromosome, suggesting they share a bidirectional promoter (Fig. S2A). Previous studies have highlighted the potentially toxic nature of the product of HDS^47,48^, which is detoxified in the subsequent enzymatic step by HDR^47,48^, making it particularly interesting to study both enzymes together. To our knowledge, this is the first report suggesting that Mp*HDS* and Mp*HDR* genes may be regulated by a shared promoter region. Initial experiments revealed that the 2 kb region upstream of Mp*HDR* was insufficient to drive expression, whereas the 1.9 kb region upstream of Mp*HDS* was adequate (Fig. S2B). Expression of a construct driving Mp*HDS-eGFP* and Mp*HDR-mVenus* bidirectionally (Fig. S2C) demonstrated that, like other MEP enzymes, both proteins localized to plastids (Fig. 1C, D). Their expression was stronger, if not exclusive, to OB cells in Day 0 gemmae (Fig. 1C, D), before becoming more widespread across tissues at later developmental stages (Fig. S1A, B).

Finally, we analyzed the subcellular localization and cell-type expression of the enzyme forming the linear diterpene precursor geranylgeranyl pyrophosphate (GGPP)^49,50^. Among the IDS candidates^27^, MpIDS2 and MpIDS3 share 68% and 62% protein homology, respectively, with GERANYLGERANYL PYROPHOSPHATE SYNTHASE 11 (GGPPS11) from *Arabidopsis thaliana*, the most highly expressed and functionally dominant GGPPS in *Arabidopsis*^51^, which operates as a homodimer^52^. However, transcriptomic data revealed that Mp*IDS3* exhibited consistently low expression across all tissues and developmental stages^12–14^. The other IDS candidates, MpIDS4 and MpIDS5, share 71% and 57% protein homology with SOLANESYL PYROPHOSPHATE SYNTHASE and GERANYL PYROPHOSPHATE SYNTHASE, respectively, suggesting potential roles in ubiquinone biosynthesis^53^ (MpIDS4) and trans-monoterpene precursor formation (MpIDS5). MpIDS2 emerged as the strongest candidate for the primary GGPPS in *Marchantia*, given its higher sequence homology and stronger expression. We therefore re-annotated it as MpGGPPS (Table 1 and S1). The translational fusion to Mp*GGPPS* showed localization to plastids in all cell types, with particularly strong expression in OB cells (Fig. 1E).

For the MVA pathway and sesquiterpene synthesis, we examined the rate-limiting enzyme 3-HYDROXY-3-METHYLGLUTARYL-COA REDUCTASE (MpHMGR*)*^54^ (Table 1). Expression of _*Pro*_Mp*HMGR:*Mp*HMGR-mVenus* revealed a restricted localization to oil body cells in gemmae (Fig. 1G) and 14-day-old plants (Fig. S1D). The enzyme appeared to display a dual localization pattern: a punctate distribution around the OB, indicative of Golgi, and a web-like pattern surrounding the nucleus, consistent with ER localization (Fig. 1G). As a key enzyme downstream of the MVA pathway, we analyzed FARNESYL PYROPHOSPHATE SYNTHASE (MpFPS) (Table 1), which forms farnesyl pyrophosphate (FPP)^55^, the linear precursor to all sesquiterpenes. Annotated as IDS1^27^ in the Marchantia.info database, MpFPS shares 66% sequence similarity with both FPS homologs from *Arabidopsis thaliana*. Expression analysis revealed that MpFPS is specifically expressed in OB cells, persisting even after 14 days (Figs. 1F and S1E). Contrary to the prediction of mitochondrial localization by DeepLoc 2.1^28^ (Table 1), the enzyme appeared predominantly cytosolic, as indicated by its uniform signal surrounding the OB and delineating the plastids (Fig. 1F).

**Fig. 2:**
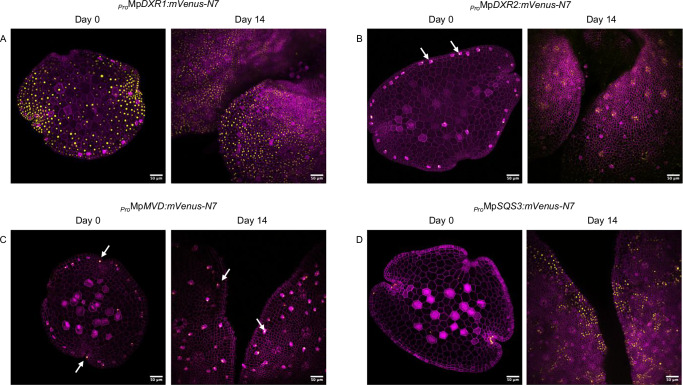
Confocal imaging of transcriptional reporters for additional isoprenoid biosynthetic genes in *Marchantia polymorpha.* **A**_*Pro*_Mp*DXR1:mVenus-N7*, **B**_*Pro*_Mp*DXR2:mVenus-N7*, **C**_*Pro*_Mp*MVD:mVenus-N7*, and **D**_*Pro*_Mp*SQS3:mVenus-N7*. The mVenus signal (yellow), localized to the nucleus, indicates the activation of these promoters in specific cell types, while mScarlet fluorescence (purple) delineates cellular boundaries. Each construct is represented by two panels: day 0 gemmae (left, scale bar: 50 μm) and the meristem area of 14-day-old plants (right, scale bar: 50 μm). White arrows indicate mVenus signals detected in oil body cells.

We further investigated the expression pattern^56^ of the promoters driving the genes encoding *1-deoxy-D-xylulose 5-phosphate reductoisomerases*^57,58^ (Mp*DXR1* and Mp*DXR2*) and *mevalonate diphosphate decarboxylase* (MpMVD)^59^ using transcriptional reporters. The corresponding enzymes, MpDXRs and MpMVD, catalyze the second enzymatic step of the MEP pathway and the penultimate enzymatic step of the MVA pathway, respectively (Table 1). _*Pro*_Mp*DXR1:mVenus-N7* showed ubiquitous expression (Fig. 2A), whereas _*Pro*_Mp*DXR2:mVenus-N7* showed specificity to OB cells in Day 0 gemma (Fig. 2B). Similarly, _*Pro*_Mp*MVD:mVenus-N7* exhibited specificity to OB cells, even in 14-day-old thalli (Fig. 2C).

Finally, given that the expression of some MVA biosynthetic genes and Mp*FPS* appears specific to OB cells, we investigated whether this pattern extends to phytosterol biosynthesis. Phytosterols are essential structural components of plant membranes^60^ and are initially formed through the fusion of two FPP molecules to produce squalene, a reaction catalyzed by SQUALENE SYNTHASE^61^ (SQS). We therefore examined the transcriptional reporter of the Mp*SQS* promoter. Among the three squalene synthase-like genes annotated in the *Marchantia* genome (Mp*SQS1-3*) (Table 1 and S1), we selected Mp*SQS3* based on its higher protein sequence homology (60%) with *Arabidopsis thaliana* SQUALENE SYNTHASE. In contrast, MpSQS1 showed greater similarity (73%) to *Arabidopsis* PHYTOENE SYNTHASE, suggesting a role in carotenoid biosynthesis, while MpSQS2 shared 52% homology with an uncharacterized terpenoid synthase. Plants expressing the _*Pro*_Mp*SQS3:mVenus-N7* constructs show a mVenus signal in meristematic regions in Day 0 gemmae, followed by ubiquitous expression in later developmental stages (Fig. 2D). These findings indicate that sterol biosynthesis is likely to occur broadly throughout the plant, based on the widespread activity of the MpSQS3 promoter.

Although localization data reflect relative protein accumulation rather than absolute presence or absence, our findings provide a comprehensive map of the subcellular localization and expression patterns of key terpene biosynthetic enzymes, offering insights into the spatial organization of biosynthetic intermediates and terpene scaffolds in *Marchantia polymorpha*.

### Exogenous production of taxadiene and β-amyrin in *Marchantia polymorpha* whole plants *vs*. oil body cells

Given the observed expression of terpene precursor enzymes predominantly in OB cells, we aimed to harness the metabolic potential of these specialized structures. As proof of concept, we attempted to produce the diterpene taxadiene^29^, and the triterpene β-amyrin^32^.

We generated constructs to express *Taxus baccata taxadiene synthase* (*TXS*)^62^ or *Talinum paniculatum β-amyrin synthase*^63^ (*β-AS*), driven by either the constitutive _*Pro*_*UBE2* or the OB cell-specific Mp*R2R3-MYB2* promoter (_*Pro*_*MYB2*)^37^, enabling expression throughout the entire plant or specifically within OB cells, respectively. TXS catalyzes the cyclization of the linear diterpene precursor GGPP into taxadiene, while β-AS converts the linear triterpene precursor 2,3-oxidosqualene into β-amyrin.

**Fig. 3:**
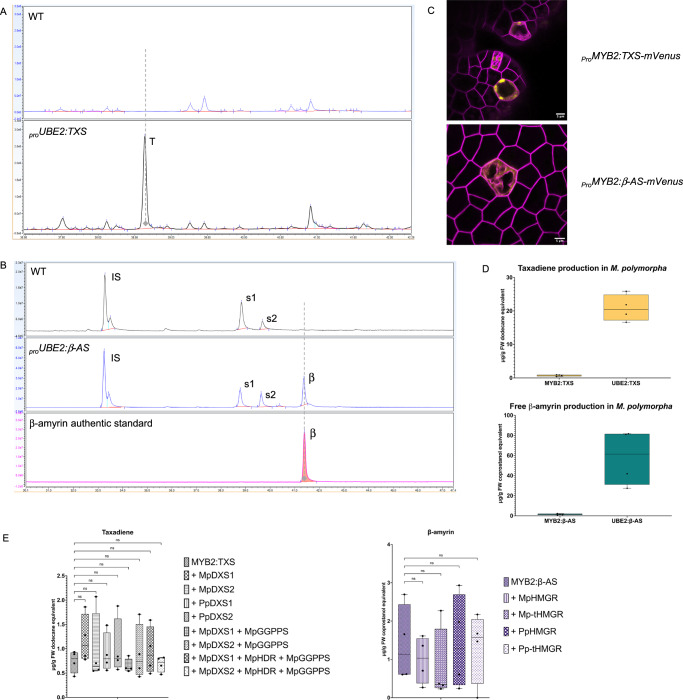
Production of taxadiene and β-amyrin in *Marchantia polymorpha* and subcellular localization of their synthases. **A** Total ion chromatograms (TICs) of terpene extracts from wild-type (WT, upper panel) and transgenic plants expressing _*Pro*_*UBE2:TXS* (*taxadiene synthase*, lower panel), highlighting the taxadiene peak (T). **B** TICs of trimethylsilyl-derivatized terpene extracts, showing WT (upper), a transgenic line expressing _*Pro*_*UBE2:β-AS* (*β-amyrin synthase*, middle), and an authentic β-amyrin standard (lower). Peaks: IS (internal standard: coprostan-3-ol), s1 (campesterol), s2 (stigmasterol), and β (β-amyrin). **C** Subcellular localization of TXS and β-AS fused to mVenus under the _*Pro*_*MYB2* promoter, showing plastid localization for TXS (upper panel) and ER or cytosol localization for β-AS (lower panel). mScarlet fluorescence (purple) delineates cellular boundaries. Scale bar: 5 μm. **D** Quantification of taxadiene (upper graph) and β-amyrin (lower graph) in plants expressing _*Pro*_*UBE2:TXS* and _*Pro*_*UBE2:β-AS*, respectively (whole-plant expression) or _*Pro*_*MYB2:TXS* and _*Pro*_*MYB2:β-AS* respectively (oil body cell-specific expression). Data represent four independent lines per condition. **E** Quantification of taxadiene (left) and β-amyrin (right) in transgenic lines overexpressing precursor genes. For taxadiene, combinations included Mp*DXS1*, Mp*DXS2*, Pp*DXS1*, Pp*DXS2*, Mp*HDR*, and Mp*GGPPS*. For β-amyrin, Mp*HMGR*, Mp*-tHMGR*, Pp*HMGR*, and Pp*-tHMGR* were tested. Box plots represent data points from independent transformants. One-way ANOVA with Dunnett's multiple comparison tests (*p* < 0.05) showed no significant differences (ns) across gene combinations.

In plants expressing _*Pro*_*UBE2:TXS*, we detected a prominent new peak in hexane extracts (Fig. 3A), corresponding to taxadiene based on its mass spectrum (MS), as described in prior publications^64,65^. While no commercial taxadiene standards were available, we identified the compound by its characteristic ion at m/z 122 and the molecular ion [M + ] at m/z 272 (Fig. S3A). Quantification of taxadiene in four independent transformants expressing either _*Pro*_*UBE2:TXS* or _*Pro*_*MYB2:TXS* revealed significantly higher levels of taxadiene in whole plants compared to those with expression restricted to OB cells (Fig. 3D). Specifically, _*Pro*_*UBE2:TXS* lines produced ~21 µg of taxadiene per g of fresh weight (FW), whereas _*Pro*_*MYB2:TXS* lines produced only 0.7 µg/g FW (Fig. 3D, E), dodecane equivalent. The correct targeting of TXS protein in OB cells was confirmed using a _*Pro*_*MYB2:TXS-mVenus* fusion construct, which showed plastid localization (Fig. 3C).

Similarly, plants expressing _*Pro*_*UBE2:β-AS* produced a new compound, identified as β-amyrin through its MS profile and comparison with an authentic standard (Figs. 3B and S3B). The _*Pro*_*UBE2:β-AS* lines produced an average of 58 µg of β-amyrin per g of fresh weight (coprostanol equivalent), whereas lines expressing the enzyme specifically in OB cells yielded an average of 1.5 µg/g FW (Fig. 3D, E). In MYB2-driven *β-AS-mVenus* lines, the protein localized specifically in the cytosol of the OB cells (Fig. 3C).

These data indicate that precursors are still present in non-OB cells and contribute to higher terpene yields in plants with ubiquitous expression. Interestingly, Suire et al.^3^ reported an approximate ratio of one OB cell to 150 non-OB cells in gemmae. Whether this ratio is maintained in older thalli remains unknown, as this would require measuring cell density in depth. Despite this uncertainty, our quantitative data show that OB-specific expression results in yields only ~30-fold lower for taxadiene and ~40-fold lower for β-amyrin, rather than a 150-fold decrease, which could hypothetically suggest that OB cells may have a higher precursor availability or storage capacity compared to the non-OB cells.

However, possible higher precursor availability in OB cells does not necessarily preclude flux limitations during engineered terpene biosynthesis. To investigate whether precursor supply was constraining the production of taxadiene and β-amyrin in these cells, we co-expressed *TXS* and *β-AS* with key rate-limiting enzymes of the MEP and MVA pathways (i.e., Mp*DXS* and Mp*HMGR*, respectively), as our work demonstrated that many of these enzymes appeared to be highly expressed in OB cells.

For taxadiene production via the MEP pathway, we co-expressed Mp*DXS1* or Mp*DXS2* with *TXS*. To mitigate potential homology-dependent gene silencing associated with overexpressing native *Marchantia* genes^66^, we also expressed the homologous Pp*DXS1* and Pp*DXS2* genes from *Physcomitrium patens*, which have recently been re-annotated as Pp*DXS1A* and Pp*DXS1D*^67^. To further enhance precursor flux, we introduced Mp*HDR* and Mp*GGPPS*, two key enzymes previously shown to increase GGPP flux for diterpene production in *Nicotiana benthamiana*^68,69^.

For β-amyrin production through the MVA pathway^70^, we overexpressed the *HMGR* genes from *Marchantia* (Mp*HMGR*) and *Physcomitrium* (Pp*HMGR*), alongside truncated versions designed to remove their negative feedback regulatory domain^71,72^, thereby increasing the pool of terpene precursors and allowing unrestrained production of β-amyrin. These truncated variants (Mp*-tHMGR* and Pp*-tHMGR*) lack the first 148 and 146 amino acids, respectively, thereby removing both transmembrane domains. This truncation strategy follows the routinely used tHMGR design for triterpene production in *N. benthamiana*^73^. All genes were expressed under the MYB2 promoter to specifically target OB cells.

Quantitative analysis of four independent transformants showed no significant increase in taxadiene or β-amyrin levels upon overexpression of these precursor supply genes (Fig. 3E). We confirmed the correct targeting to OB cells and subcellular localization of *Marchantia DXS*, *HDR*, *GGPPS*, *HMGR*, and *tHMGR* by fusing these genes with mVenus under the control of the MYB2 promoter (Fig. S4). MpDXSs, MpHDR, and MpGGPPS enzymes specifically localized to plastids of the OB cells (Fig. S4A–D). However, their overexpression to increase precursor availability for TXS did not result in increased taxadiene levels, nor in the level of main endogenous sesquiterpenes (Supplementary Data 1 and 2), which were tentatively identified by MS library matching and by comparison of Kovats retention indices^74^ (Fig. S5 and Table S3). While Mp-tHMGR-mVenus appeared to localize in the ER and Golgi apparatus of OB cells (Fig. S4E), the native version driven by the MYB2 promoter remained undetectable. To address this, we re-cloned Mp*HMGR* under the 2x35S promoter and successfully detected the protein, which accumulated exclusively in the ER of OB cells (Fig. S4F). Interestingly, when the truncated version (Mp-tHMGR) was expressed under the 2x35S promoter, the fusion protein showed expression across all cells (Fig. S4G), suggesting that the negative feedback domain removed in this construct may play a role in restricting localization to OB cells.

Overall, these results demonstrate that overexpression of selected rate-limiting enzymes was insufficient to significantly enhance yields of exogenous terpenes in *Marchantia* OB cells. This could reflect either that precursor availability was already sufficient to saturate the reactions or that additional regulatory mechanisms may play critical roles in terpene biosynthesis and accumulation in these specialized cells.

### Accumulation of terpenes in OBs may require a specific transporter

Although overexpression of presumed rate-limiting enzymes is a standard strategy in terpene engineering, in our hands, it did not enhance exogenous terpene yields when targeted to OB cells. Our initial aim was to produce exogenous terpenes and rely either on their putative passive diffusion into the OB lumen or on the earlier hypothesis that biosynthetic enzymes positioned at the OB membrane, which we assumed could allow newly formed terpenes to enter the lumen. However, after determining the subcellular localization of the enzymes, we realized that these compounds may not naturally accumulate in OBs: taxadiene may primarily localize to plastids, while β-amyrin could accumulate in the cytosol. Furthermore, previous studies demonstrated high and exclusive expression of an Mp*ABCG1* gene in oil body cells^35^, with its protein localized to the OB membrane when expressed under its native promoter, and to the plasma membrane of other cells when driven by a non-OB-specific promoter^7^. This prompted us to hypothesize that precursor flux in *Marchantia* predominantly supplies sesquiterpene biosynthesis in the oil body cells and that a specific transporter, potentially MpABCG1, may be required, directly or indirectly, for their accumulation inside OBs.

**Fig. 4:**
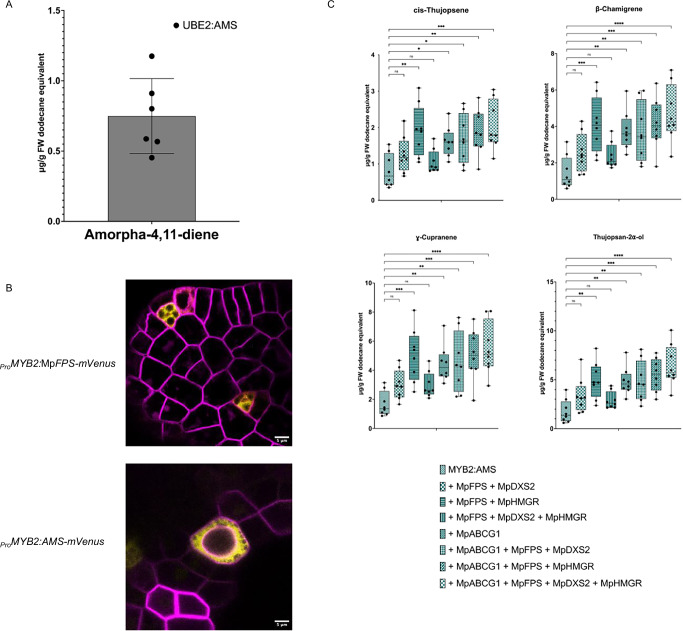
Expression of amorphadiene synthase (*AMS*) in *Marchantia polymorpha* with or without precursor supply genes and the MpABCG1 transporter, and its effect on endogenous sesquiterpene levels. **A** Quantification of amorpha-4,11-diene levels in plants expressing *Artemisia annua* AMS under the *Marchantia UBE2* promoter. The compound was detected in six independent transformants (mean ± SD; *n* = 6). **B** Subcellular localization of MpFPS and AMS proteins when expressed under the oil body-specific *MYB2* promoter. mVenus fluorescence (yellow) indicates protein localization, with signals observed in oil body cells, while mScarlet fluorescence (purple) marks cell boundaries. Scale bars: 5 μm. **C** Quantification of endogenous sesquiterpenes, including cis-thujopsene, β-chamigrene, γ-cuprenene, and thujopsan-2α-ol, in lines expressing Aa*AMS* with or without precursor supply genes (Mp*FPS*, Mp*DXS2*, and/or Mp*HMGR*) and with or without Mp*ABCG1*, under _*Pro*_*MYB2*. Box plots represent data points from eight independent transformants (*n* = 8), with significance determined by one-way ANOVA followed by Dunnett's multiple comparison test (*p < 0.05; **p < 0.01; ***p < 0.001; ****p < 0.0001; ns  not significant).

To investigate whether MpABCG1 could facilitate the accumulation of any sesquiterpenes in *Marchantia*’s OBs, we expressed *Artemisia annua amorphadiene synthase* (*AMS*), a sesquiterpene synthase that converts FPP into amorpha-4,11-diene^34^. Cyclisation of FPP into this sesquiterpene likely occurs via the bisabolyl cation^75^, similar to the endogenous β-chamigrene^76^, therefore we expected this structural similarity to enable amorpha-4,11-diene transport into OBs. We targeted *AMS* expression to either the whole plant (_*Pro*_*UBE2*) or specifically in OB cells (_*Pro*_*MYB2*). Additionally, we co-expressed *AMS* and Mp*ABCG1* in OB cells, alongside Mp*HMGR*, Mp*DXS2*, and Mp*FPS*, with FPS enzyme often referred to as a rate-limiting enzyme in sesquiterpene biosynthesis^77^, or alternatively as a key step in substrate channeling toward FPP. By introducing MpABCG1 and MpFPS, we aimed to overcome potential transport limitations and to test whether this combination could further increase FPP availability for sesquiterpene production. Amorpha-4,11-diene was detected only in transgenic lines expressing the construct _*Pro*_*UBE2:AMS* (Fig. S6), with an average yield of 0.75 µg/g FW in six out of eight independent transformants (Fig. 4A, Supplementary data 3). No amorpha-4,11-diene was detected when expression was targeted to OB cells, either with or without precursor supply genes and Mp*ABCG1* (Fig. S6). The absence of detection is consistent with the low yields observed in whole-plant _*Pro*_*UBE2:AMS* lines, where amorpha-4,11-diene levels were nearly two orders of magnitude lower than taxadiene or β-amyrin in comparable constructs, and may therefore have fallen below the detection threshold in OB-targeted lines. Localization studies of *AMS-mVenus* and *MpFPS-mVenus* driven by the MYB2 promoter showed that both proteins localized to the cytosol of OB cells (Fig. 4B).

When measuring endogenous sesquiterpene levels in at least eight independent transformants for each gene combination, we observed that, compared to lines expressing _*Pro*_*MYB2:AMS* alone, co-expression of precursor genes significantly increased sesquiterpene levels (Fig. 4C, Supplementary Data 3). Among combinations without Mp*ABCG1*, Mp*FPS* + Mp*HMGR* boosted sesquiterpene levels the most, with a 2.8-fold average increase for the major sesquiterpenes compared to a 1.8-fold increase with Mp*FPS* and Mp*DXS2*. The overexpression of Mp*ABCG1* alongside all precursor genes led to the most significant increases in endogenous sesquiterpene levels, with a threefold increase in cis-thujopsene, a 3.2-fold increase in β-chamigrene and γ-cuprene, and a 3.6-fold increase in thujopsan-2α-ol (Fig. 4C, Supplementary Data 3). The observed increase in endogenous sesquiterpene levels when Mp*HMGR* was expressed under the MYB2 promoter also suggests that MpHMGR protein is likely localized to OB cells, despite its undetection in localization studies using the mVenus tag. This discrepancy may reflect interference from the C-terminal mVenus fusion affecting proper folding or stability of MpHMGR, whereas the native untagged version used in metabolic engineering was more efficiently expressed and active.

**Fig. 5:**
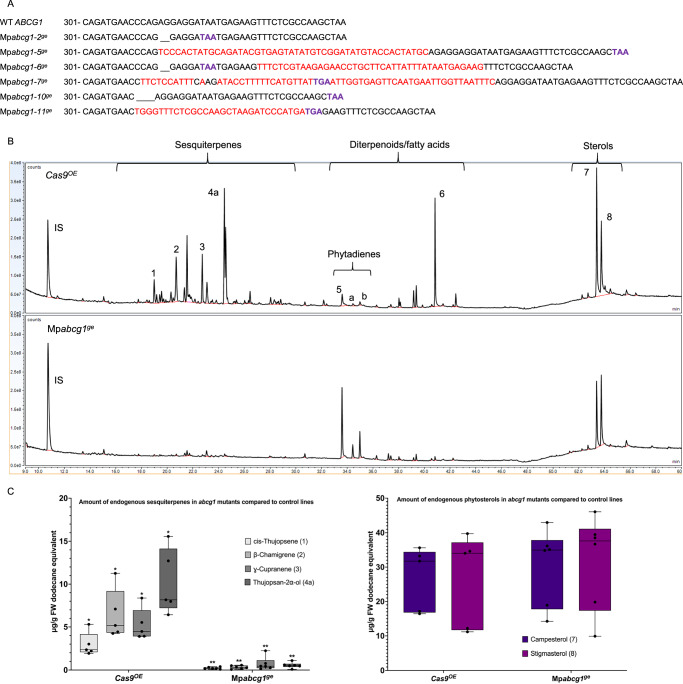
Analysis of endogenous terpene profiles in CRISPR-generated Mp*abcg1*^*ge*^ mutants compared to *Cas9*^*OE*^ controls. **A** DNA sequence alignment of the Mp*abcg1* genomic region in wild-type (WT) and CRISPR-generated Mp*abcg1*^*ge*^ mutant lines. Dashes indicate deletions, red sequences represent insertions, and purple nucleotides correspond to premature stop codons introduced by the deletions and/or insertions. **B** TICs of terpene extracts from a *Cas9*^*OE*^ control (upper chromatogram) and Mp*abcg1*^*ge*^ mutant line (lower chromatogram). Peaks corresponding to quantified compounds are labeled: sesquiterpenes (1: cis-thujopsene, 2: β-chamigrene, 3: γ-cuprenene, 4a: thujopsan-2α-ol), fatty acids/diterpenoids (5: neophytadiene, 6: phytol, a: (*Z*)-1,3-phytadiene, b: (*E*)-1,3-phytadiene), and phytosterols (7: campesterol, 8: stigmasterol). **C** Quantification of endogenous terpene levels in Mp*abcg1*^*ge*^ (*n* = 6) compared to *Cas9*^*OE*^ (*n* = 5). The left graph shows the amounts of the four major sesquiterpenes (1 to 4a) labeled in (**B**). The right graph displays the levels of phytosterol (7 and 8) labeled in (**B**). Box plots display individual data points, with bars representing the median and interquartile range. Statistical significances are denoted by asterisks (unpaired *t* tests or Welch’s tests, depending on variance equality, *p* < 0.05).

To further validate the transport role of MpABCG1, we generated CRISPR lines to disrupt its function. We obtained six independent transformants with early stop codons in the Mp*ABCG1* DNA sequence (Fig. 5A). Terpene analysis revealed a dramatic reduction or complete absence of sesquiterpenes in Mp*abcg1* mutants (Mp*abcg1*^*ge*^) compared to Cas9-only controls (*Cas9*^*OE*^) (Fig. 5B, C). Notably, this reduction was specific to sesquiterpenes, as the levels of phytosterols remained unaffected (Fig. 5B, C). Given the apparent differences in peak abundance observed in the overlaid chromatograms (Fig. 5B), we quantified additional compounds alongside neophytadiene and phytol, such as (*Z*)-1,3-phytadiene and (*E*)-1,3-phytadiene, phytol derivatives identified in prior studies^78,79^ (Fig. 5B). Quantification revealed no significant changes in these compounds when comparing the five *Cas9*^*OE*^ controls and six Mp*abcg1*^*ge*^ lines (Fig. S7).

To assess whether the reduced sesquiterpene levels in Mp*abcg1*^*ge*^ could be attributed to changes in OB characteristics, we performed BODIPY staining on three pieces of newly formed meristematic tissues from primary transformants after the second round of selection. Confocal microscopy revealed no detectable differences between Mp*abcg1*^*ge*^ lines and *Cas9*^*OE*^ in OB number (Fig. S8A) or average fluorescence intensity (Fig. S8C, Supplementary data 5). In contrast, OBs in Mp*abcg1*^*ge*^ were significantly smaller in size compared to controls (Fig. S8B, Supplementary Data 5). Since OBs in *M. polymorpha* also contain abundant non-terpenoid metabolites such as bisbibenzyls, the BODIPY signal may primarily reflect lipidic and aromatic components other than terpenes. The reduction in OB size may therefore reflect impaired terpene accumulation, which restricts OB expansion without affecting overall fluorescence intensity.

These findings suggest that MpABCG1 plays a selective role, directly or indirectly, in promoting the accumulation of endogenous sesquiterpenes, with limited evidence of an effect on other terpene subclasses or on overall OB composition.

## Discussion

In this study, we used translational and transcriptional reporters to map both the cell-type specificity and subcellular localization of enzymes involved in the early steps of terpene biosynthesis. These findings corroborate earlier transcriptomic analyses in OB-defective plants^5,7^, as well as co-expression network analysis^14^ and single-cell RNA sequencing data^35^, all of which indicate that these enzymes are OB cell-specific.

Enzymatic steps of the MEP pathway and diterpene biosynthesis, including MpDXS, MpHDS, MpHDR, and MpGGPPS, were localized to chloroplasts/plastids—referred to as chloro-amyloplasts in OB cells by Suire et al.^3^—consistent with their predicted roles in producing isoprenoid precursors for plastid-derived terpenes. In contrast, steps of the MVA pathway and sesquiterpene biosynthesis, represented by MpHMGR and MpFPS, were localized to the ER, Golgi, and cytosol, supporting their involvement in cytosolic terpene precursor production. With the exception of MpFPS, all studied enzymes localized as predicted by DeepLoc 2.1^28^. However, our experimental conditions allowed us to localize steady-state accumulation of proteins and did not account for any dynamic behavior due to protein trafficking^80^. Our findings corroborate earlier studies^3^ that reported high or exclusive expression of isoprenoid biosynthetic enzymes in OB cells. Given that OBs are formed through redirection of the secretory pathway^7^, some enzymes like MpHMGR—which localizes to both the ER and Golgi depending on the OB cell or plant being imaged—may transit to other compartments during OB development, supporting the possibility of their transient presence around the OB membrane^3^. For each translational or expression pattern reporter, we selected promoter lengths based on a single ATAC-seq^38^ peak, ensuring coverage of the putative core promoter and upstream regulatory regions. In some cases, promoters (including the 5'UTR) were shorter than the typically advised 2.5 kb length^37,41^, balancing the need for regulatory coverage with constraints related to sequence synthesis. For instance, a promoter as short as 970 bp like _*Pro*_Mp*GGPPS* was sufficient to drive strong and ubiquitous signal in chloro-amyloplasts. However, in other instances, such as _*Pro*_Mp*DXR2*, promoter functionality required extending the length from 1.7 kb to 2.7 kb to achieve detectable expression. Altering promoter lengths could include or exclude positive or negative regulatory elements affecting cell-type specificity; therefore, further systematic studies would be needed to draw concrete conclusions about the relationship between promoter structure and regulatory outcomes. Nevertheless, our results confirm the specialized role of OB cells in terpene biosynthesis and highlight the importance of carefully selecting promoters to ensure accurate expression and localization of biosynthetic enzymes.

With respect to precursor supply, our study suggests that the MVA pathway predominantly provides precursors for sesquiterpene synthesis in *Marchantia polymorpha*, supported by the apparent exclusive expression of MpHMGR and MpMVD in OB cells and the significant increases in endogenous sesquiterpene levels observed when Mp*HMGR* and Mp*FPS* were co-expressed. Notably, MpFPS—also a potential OB-specific enzyme—emerged as a critical limiting step in sesquiterpene biosynthesis, consistent with previous findings in *Nicotiana tabacum*^81^. The MEP pathway’s contribution to sesquiterpene synthesis cannot be ruled out, given the specific expression of Mp*DXS2* and Mp*DXR2* in OB cells and the fact that a previous study using GC-MS analysis of physically extracted OB content detected sesquiterpenes^2^ as the only subclass of terpenes. If OBs also store monoterpenes like limonene^26^ or specific diterpenes, their levels remained undetectable or unchanged despite the targeted expression of precursor supply genes. Furthermore, overexpression of key MEP genes and Mp*GGPPS* had no effect on taxadiene yield, despite co-localization of these enzymes with TXS in plastids. This suggests that a significant portion of the precursors is redirected toward sesquiterpene synthesis instead. Supporting this, co-expression of Mp*DXS2* with Mp*FPS*, Mp*HMGR*, and Mp*ABCG1* mildly increased the sesquiterpene levels compared to the same combination of genes without Mp*DXS2*, demonstrating a modest effect of Mp*DXS2* on sesquiterpene yield. Dual contribution from the MVA and MEP pathways in *Marchantia polymorpha* would parallel findings in *Artemisia annua*, where inhibitor assays demonstrated a mixed origin of precursors^82^. In contrast, Mp*DXS1* and Mp*DXR1* are ubiquitously expressed and likely play broader roles in synthesizing essential metabolites, such as phytyl diphosphate for chlorophyll production. Supporting this, ChIP-seq studies in *Marchantia* have shown that the chloroplast biogenesis regulator GLK binds to the promoter of Mp*DXR1*^83^, further highlighting its role in primary metabolism rather than specialized metabolite production. This divergence in expression patterns between Mp*DXS1* and Mp*DXS2* is consistent with the functional distinction between Class I and Class II *DXS* isoforms, typically associated with primary and secondary metabolism, respectively^84^. The presence of such functional specialization in an early-diverging land plant is in line with recent findings in *Physcomitrium patens*^67^. The specific localization of MpHMGR, MpMVD, and MpFPS protein fusions in OB cells raises intriguing questions about how essential metabolites, such as sterols, are produced in non-OB cells. Despite the ubiquitous expression of squalene synthase Mp*SQS3*, which catalyzes the formation of squalene as a precursor to sterols, the source of precursors for sterol synthesis in non-OB tissues remains unclear. Notably, sterol levels in Mp*abcg1*^ge^ were unaffected, suggesting that OBs are not the primary site of sterol synthesis or storage for the rest of the plant. This is further supported by the observation that sterol levels remain unchanged in plants defective in OB cell differentiation^5^. This raises the possibility that other, as-yet-unannotated MVA enzymes may supply precursors for sterol biosynthesis in non-OB cells. Alternatively, it is plausible that the genes studied here are expressed ubiquitously but accumulate at levels too low to be detected in other cell types with our confocal microscopy studies. These findings highlight the complexity of precursor flux and compartmentalization in *Marchantia*, accentuating the need to further investigate how primary metabolite synthesis is maintained across tissues.

**Fig. 6:**
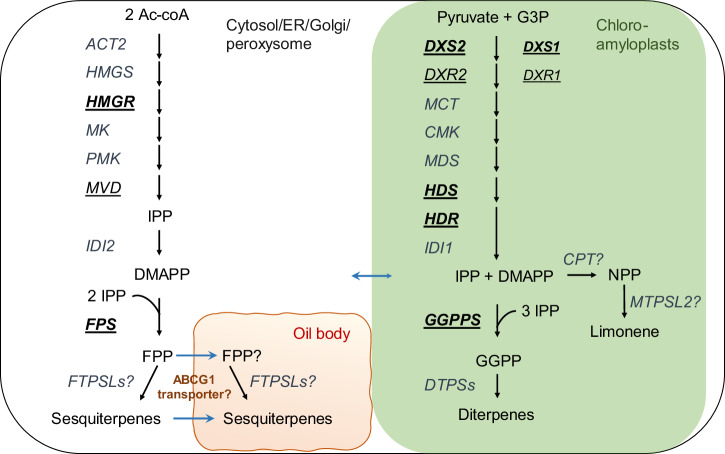
Proposed overview of the isoprenoid precursor pathways in *Marchantia polymorpha* oil body cells based on previous works and findings from this study. Biosynthetic scheme illustrating the mevalonate (MVA) and methylerythritol phosphate (MEP) pathways in *Marchantia polymorpha*. Enzymes shown in bold and underlined were localized using translational reporters, confirming their subcellular localization. Enzymes shown in underlined text only were analyzed with promoter expression constructs, indicating activity in oil body cells. Details of enzymatic steps and annotations can be found in Table 1 and Table S1. Abbreviations: Ac-CoA, acetyl coenzyme A; G3P, glyceraldehyde-3-phosphate; CPT, cis-prenyl transferase; MTPSL2, microbial terpene synthase-like 2, functionally characterized in yeast as a limonene synthase (Kumar et al.).

Finally, our findings point to an important role for the ABC transporter MpABCG1 in determining terpene accumulation within oil body cells. Our study demonstrated that exogenous terpenes, such as amorpha-4,11-diene, taxadiene, and β-amyrin, could be produced throughout the whole plant. However, achieving useful yields within OB cells proved less straightforward, even with the overexpression of key biosynthetic genes. This led us to consider whether transport into OBs might be a limiting factor for the accumulation of exogenous compounds. Supporting this, CRISPR-mediated disruption of Mp*ABCG1* resulted in a sharp reduction in endogenous sesquiterpene levels, demonstrating that MpABCG1 plays a crucial role in terpene accumulation. However, whether MpABCG1 directly transports sesquiterpenes or acts indirectly to facilitate their accumulation in the OB lumen remains unclear, and its exact function therefore remains an open question. Some ABCG transporters have been experimentally characterized through transport assays to identify their specific substrates^85^. Among those sharing higher protein sequence similarity with MpABCG1 (~55%), some exhibit broad substrate specificity, such as *Nicotiana tabacum* NtPDR1, which can accommodate chemically diverse terpenes like the sesquiterpenes sclareol and capsidiol, and the diterpene cembrene^86^. In contrast, others are more selective, such as *Arabidopsis thaliana* ABCG29, which transports only *p*-coumaryl alcohol^87^ or *Oryza sativa* ABCG36, which exports cadmium and cadmium conjugate^88^. This raises the question of whether MpABCG1 could directly transport the 35+ endogenous sesquiterpenes detected by our GC-MS analysis and previous studies^2^—a number that likely underestimates the total—as these methods primarily detect compounds with limited hydroxylation. An alternative hypothesis is that MpABCG1 may influence the transport of FPP into the OB lumen rather than that of the sesquiterpene end-products. Supporting this, we observed that a translational reporter of the *fungal terpene synthase-like 2* (*FTPSL2*), an endogenous sesquiterpene synthase characterized by Kumar et al.^26^, was detected within the OB lumen in two independent lines, but only in one lobe out of four in 14-day-old plants (Fig. S9). Because fluorescent protein fusions may occasionally affect trafficking, targeting or organelle morphology^1,89–91^, these technical factors cannot be fully excluded as contributors to the restricted MpFTPSL2–mVenus signal. Nevertheless, the observed localization provides preliminary support for OB lumen functioning as a biosynthetic compartment^3^ rather than solely storage, while the limited localization pattern detected underscores the need for further confirmation. To date, no ABC transporter has been reported to transport phosphorylated isoprenoid intermediates. Indeed, previous research demonstrated that the transport of shorter-chain prenyl diphosphates, such as IPP, occurs across chloroplast membranes via a proton-symport mechanism rather than an ATP-dependent ABC transporter system^92^. However, the OB membrane cannot be directly compared to the chloroplast double membrane, and FPP, being three times larger than IPP, may have different transport requirements. Interestingly, FPP shares amphiphilic properties and a sesquiterpene backbone with abscisic acid (ABA), a known substrate of several ABCG transporters^85^. For example, *Triticum aestivum* ABCG36^93^ and *Arabidopsis* ABCG40^94^, both sharing ~45% protein sequence similarity with MpABCG1, are specific ABA transporters. Moreover, the ability of *Arabidopsis* ABCC5 to transport inositol hexakisphosphate^95^ suggests that phosphate-containing metabolites can indeed be substrates for ABC-type transporters. If this provisional hypothesis proves correct, it would significantly reshape strategies for engineering terpene biosynthesis in OBs. In this scenario, prenyl phosphate precursors would need to be transported into the OB lumen, and terpene synthases precisely targeted to the lumen or OB membrane to enable localized production of desired compounds. In line with this, we present a tentative map of terpene biosynthesis in *Marchantia* OB cells (Fig. 6). This map summarizes our findings on the potential accumulation and subcellular localization of key enzymes involved in terpene precursor synthesis, and integrates speculative elements regarding the remaining MVA and MEP pathway steps within OB compartments, as well as the putative role of MpABCG1 (Fig. 6). Overall, our work provides a comprehensive view of terpene synthesis in *Marchantia polymorpha* OB cells by corroborating predicted and experimentally observed subcellular localization of key isoprenoid biosynthetic enzymes. While the exact role of MpABCG1 remains to be fully elucidated, our results suggest that this transporter is involved in the accumulation of endogenous sesquiterpenes and highlight its potential relevance in metabolic compartmentalization. Our study also clarifies which engineering strategies may be limited in *Marchantia*, particularly when targeting the production of exogenous terpenes in OBs. Beyond *Marchantia*, these insights offer a foundation for developing more targeted approaches to achieve efficient terpene biosynthesis in specialized plant compartments.

## Methods

### Plant material and growth conditions

*Marchantia polymorpha* subspecies *ruderalis* accessions Cam-1 (male) and Cam-2 (female) were used in this study^96^. Plants were cultivated on solid 0.5× Gamborg B–5 basal medium (#G398, PhytoTech Labs, Lenexa, Kansas, USA) adjusted to pH 5.7–5.8 and solidified with 1.2% (w/v) agar micropropagation grade (#A296, PhytoTech Labs). They were maintained under continuous light at 22 °C with a light intensity of 150 μmol/m^2^/s. For general propagation of the lines, plants were grown in 94 × 16 mm Petri dishes (#633181, Greiner Bio-One, Kremsmünster, Austria). For imaging purposes, plants were cultivated on gridded 65 × 14.5 mm Gosselin™ Petri dishes (#BB64-01, Corning, Corning, NY, USA). Several gemmae, originating from four independent transformants, were grown on these plates and imaged at developmental stages corresponding to Days 3, 5, 7, and 14 after germination. For gemma cup production and the collection of material for terpene analysis, the media were supplemented with 0.5% (w/v) sucrose. Plants destined for terpene extraction and quantification were grown in 100 × 25 mm Petri dishes (#D943, Phytotech Labs) containing 50 mL of medium. Spore production was carried out in 1L Microbox micropropagation containers (#O119/140 + OD119/140, Sac O_2_, Deinze, Belgium), initially maintained at 21 °C under continuous illumination (150 µmol m^−2^ s^−1^), and subsequently transferred after ~1 month to long-day conditions (16 h light/8 h dark) with the same light intensity supplemented with far-red radiation (peak emission ~730–740 nm) provided by Philips GreenPower far-red LEDs, following a previously established protocol^41^.

### Protein sequence analysis

Protein sequences of the selected MpIDS and MpSQS candidates from *Marchantia polymorpha* were analyzed using NCBI BLASTP (Basic Local Alignment Search Tool for Proteins, https://blast.ncbi.nlm.nih.gov/Blast.cgi) against the *Arabidopsis thaliana* protein database. Sequence similarity was assessed based on percentage identity and coverage to infer functional homology. To assess relatedness among ABCG transporters from different plant species, we retrieved the protein sequences of experimentally characterized ABCG with known substrate specificity from Do et al.^85^, when available. These were aligned with the MpABCG1 sequence using the UniProt^97^ online tool to evaluate percentage identity and phylogenetic grouping.

### Plasmid construction for overexpression studies

DNA fragments used to generate mVenus and metabolic constructs were synthesized by Genewiz (Azenta, Burlington, Massachusetts, USA) either as linear fragments or in pUAP1 vector^98^. Linear fragments included overhangs compatible with LguI (#ER1931, ThermoFisher Scientific, Waltham, Massachusetts, USA) cloning into L0 acceptor vectors following the Loop protocol^41^. Linear fragments and sequences synthesized in pUAP1 vectors, contained overhangs enabling direct cloning into L1 or pBy01^37^ vectors using BsaI-HF ^®^v2 enzyme (#R3733S, New England Biolabs, Ipswitch, MA, USA). Mp*FPS* and Mp*HDS* sequences were cloned from the *Marchantia* transcriptome as they did not require the removal of BsaI and LguI internal restriction sites, incompatible with the Loop cloning system. Total RNA was first extracted from *Marchantia* tissue using the RNeasy Plant Mini Kit (#74904, Qiagen, Hilden, Germany) and reverse-transcribed into cDNA with Superscript IV (#18090010, Invitrogen, Waltham, Massachusetts, USA) using random hexamer oligos (#N8080127, Invitrogen) following the protocol from the manufacturer. Target sequences were subsequently amplified from the cDNA using VeriFi® Polymerase Mix (#PB10.43-01, PCR Biosystem, London, UK). PCR products were purified using the QIAquick PCR Purification Kit (#28104, Qiagen) before being used in the cloning steps. DNA sequences exogenous to *Marchantia* were domesticated when necessary and codon-optimized using the Genewiz codon-optimization tool prior to ordering. NCBI accession numbers for each exogenous gene are as follows: *TXS* (AY424738), *β-AS* (MG492000), Pp*DXS1* (XM_024524883), Pp*DXS2* (XM_024533934), Pp*HMGR* (XM_024507706) and *AMS* (AY006482). Certain sequences were re-amplified to remove stop codons and introduce the appropriate overhangs for fusion with mVenus or eGFP tags. Primers used in this study were ordered from IDT (Integrated DNA Technology, Coralville, Iowa, USA) (Table S4). Vectors, promoters, terminators, tags, and pre-assembled cassettes used to generate the constructs were sourced from the OpenPlant toolkit^41^ or adapted from Romani et al.^37^. Final plasmids (L2, L3, or pBy01) were sequenced at Plasmidsaurus (Eugene, Oregon, USA) to confirm proper assembly and integration of all transcription units. Details of the sequences and vectors syntax are available in Supplementary data 6.

### In vitro testing of guide RNA and generation of CRISPR-based mutants

In vitro testing of candidate gRNAs was performed prior to plant transformation, following a protocol adapted from an open-access method on protocols.io^99^, with modifications described below. Genomic DNA was extracted from *Marchantia polymorpha* using the InnuPrep Plant DNA Kit (#845-KS-1060050, IST Innuscreen, Berlin, Germany). A genomic region of 860 bp containing the six candidate gRNA target sites was amplified using primers spanning from the 5′UTR to the first coding exon of the *ABCG1* sequence. For sequences of the six gRNAs and their target positions, see the table in Fig. S10A. To amplify the 860 bp genomic sequence, the forward primer AAGACTCCGGATCCGAGGG and reverse primer CCTCAACTTTGGGCAGGTCA were used. PCR amplification was performed using VeriFi® Polymerase Mix, and products were purified using the QIAquick PCR Purification Kit. For each of the six candidate gRNAs designed with the CasFinder tool^100^, a DNA template for in vitro transcription was generated by PCR. Forward primers consisted of the T7 promoter sequence, followed by the 20-nt gRNA target sequence, and the first portion of the trans-activating CRISPR RNA (tracrRNA), in the following format: GAAATTAATACGACTCACTATAGG<gRNA sequence>GTTTTAGAGCTAGAAATAGC. The reverse primer AAAAGCACCGACTCGGTGCCAC corresponded to the remaining portion of the tracrRNA scaffold. PCR templates were amplified using the single-guide acceptor CRISPR vector from Sauret-Guëto et al.^23^ containing the gRNA scaffold. PCR products were purified using the QIAquick PCR Purification Kit and quantified prior to in vitro transcription. PCR templates for each of the six candidate gRNAs were transcribed into RNA using the MEGAshortscript™ T7 Transcription Kit (#AM1354, ThermoFisher Scientific) following the manufacturer’s protocol, including treatment with TURBO DNase. RNA was precipitated by adding 15 µL RNase-free water and 15 µL LiCl Precipitation Solution (#10498254, Fisher Scientific) and processed according to the manufacturer’s instructions. In vitro cleavage reactions were performed using the Cas9 Nuclease (#M0646T, New England Biolabs) following the supplier’s protocol, with 300 nM in vitro–transcribed sgRNA and 200–300 ng of the genomic 860 bp amplicon as substrate. Reactions were incubated at 37 °C for 1 h, then treated with 1 µL Proteinase K (#11588916, Fisher Scientific) at 65 °C for 10 min. Cleavage products were resolved immediately on an agarose gel alongside a 100 bp DNA ladder (#N3231S, New England Biolabs). Among the six tested guides (Fig. S10A), gRNA #1 showed a slightly reduced intensity of the full-length 860 bp band (Fig. S10B), suggesting possible in vitro activity, whereas gRNA #5 produced a clear cleavage pattern with two distinct fragments and a reduced intensity of the 860 bp band. Both gRNAs #1 and #5 were cloned into the single-guide acceptor vector following Sauret-Guëto et al. protocol^23^. Numerous mutants were obtained only with gRNA #5, while no mutants were recovered for gRNA #1.

### *Agrobacterium*-mediated transformation of *Marchantia* spores and selection of independent transformants

*Marchantia* spores were sterilized and transformed following previously described protocols, with slight modifications. Briefly, a single archegoniophore, dried and stored in silica beads, was mixed in 1.5 mL of a chlorinated solution consisting of one Milton sterilizing tablet (troclosene sodium; Boots, UK) dissolved in 10 mL of sterile water. Spores were released into the solution, filtered through a 40 µm cell strainer (#542040, Greiner Bio-One) and incubated in the sterilizing solution for 30 min. After sterilization, spores were harvested by centrifugation, resuspended in sterile water, and spread on Gamborg medium agar plates (without sucrose). After 5 days of germination, the sporelings were co-cultured with *Agrobacterium tumefaciens* GV3101 carrying the constructs of interest. Transformation of *Agrobacterium* with the constructs was performed using the freeze–thaw method^101^. Sporelings and *Agrobacterium* were co-cultured for 2 days in a 4 mL solution of 0.5× Gamborg B–5 plus supplements, as previously described^41^, and incubated at 22 °C under continuous shaking and light. Following co-culture, sporelings were collected on 70 µm cell strainers (#542070, Greiner Bio-One), rinsed thoroughly with sterile water, and plated on 90 mm Petri dishes containing 0.5× Gamborg B5 medium supplemented with 100 µg/mL cefotaxime (#BIC0111, Apollo Scientific, Bredbury, UK) to eliminate *Agrobacterium* and 20 µg/mL hygromycin (#10687010, Invitrogen) to select for transformants. After two weeks on the first selective plates, eight transformants were typically transferred to a second plate containing the same selection agents to confirm successful transformation and eliminate residual *Agrobacterium*. For lines expressing fluorescent proteins (mScarlet, mVenus or eGFP), transformants were pre-selected under a Leica stereo microscope (#MDG41, Leica, Wetzlar, Germany) using suitable filter sets to confirm the presence of fluorescence signals. For lines expressing exogenous terpenes, eight plants were randomly chosen, grown for one month on the second selective plates, and subsequently extracted to confirm the presence of the terpene of interest. The four best lines producing the desired terpene were then transferred to sucrose-supplemented plates to quantify endogenous and exogenous terpene levels. For lines co-expressing *AMS* and precursor supply genes, a single vector carrying up to six transcription units could not be constructed due to cloning or vector size limitations. Attempts to co-transform using two *Agrobacterium* strains carrying distinct plasmids—one with hygromycin B as the selection agent and the other with chlorsulfuron—resulted in an insufficient number of transformants. Therefore, transformants were selected based on hygromycin B resistance for *AMS* (with or without Mp*ABCG1*) on one vector, and the presence of an mScarlet-Lti6b fluorescent signal from the second vector carrying Mp*FPS*, Mp*HMGR*, and/or Mp*DXS2*. All eight independent lines were retained to measure amorphadiene and endogenous terpene levels. To genotype CRISPR-generated Mp*abcg1*^*ge*^ mutants and *Cas9*^*OE*^ lines, genomic DNA was extracted using the InnuPrep Plant DNA kit. For Mp*abcg1*^*ge*^ lines, a 500 bp DNA fragment spanning the gRNA target site was amplified using Q5® High-Fidelity DNA Polymerase (#M0491S, New England Biolabs). The resulting PCR products were purified and sequenced by Genewiz to confirm the disruption or not of the Mp*ABCG1* coding sequence. The presence of the Cas9 cassette in control lines was confirmed by amplification with specific primers (listed in Table S4) and subsequent sequencing. A total of 16 lines were genotyped for the Mp*abcg1*^*ge*^ ones, and eight for the *Cas9*^*OE*^ controls.

### Laser scanning confocal microscopy

For transgenic lines expressing fluorescent proteins, images were acquired on an upright Leica SP8X confocal microscope equipped with a 460–670 nm supercontinuum white light laser, two continuous wavelength laser lines of 405 nm and 442 nm and a five-channel spectral scanhead (four hybrid detectors and one photomultiplier). Imaging was conducted using either a 25× water immersion objective (Fluotar VISIR 25×/0.95 WATER) for whole gemmae or meristematic area imaging, or a ×40 water immersion objective (HC PL APO CS2 ×40/1.10 WATER) for higher magnification images, with an additional digital zoom applied up to a factor of 5× to enhance visualization of subcellular localizations. Excitation laser wavelength and fluorescence emission bandwidth windows were as follows: 515 nm and 525–550 nm (for mVenus); 570 nm and 591–621 nm (for mScarlet); 442 nm and 645–664 nm (for chlorophyll autofluorescence). Each channel was acquired separately using a hybrid detector, with a sequential scan for each channel performed on the Leica LAS X software. To image the mVenus, eGFP and mScarlet fluorescences on the translational reporter lines carrying the bidirectional construct of Mp*HDS-eGFP* and Mp*HDR-mVenus*, excitation and emission bandwidth windows were adjusted as follows to prevent signal crosstalk: 487 nm and 497–513 nm (for eGFP); 521 nm and 530–550 nm (for mVenus); 570 nm and 588–598 nm (for mScarlet). Day 0 gemmae were imaged by mounting them on a glass slide with perfluorodecalin^102^ (#130040250, ThermoFisher) and covering them with a glass coverslip. For plants aged 3–14 days, a 15 × 16 mm gene frame (#AB0577, ThermoFisher) was placed on the glass slide to prevent compression of the plant material under the coverslip. Whole gemmae and overviews of the meristematic region in older plants were imaged using Z-stack scans, which were processed in Fiji^103^ to generate maximum intensity projections of the Z-stacks. High magnification images were acquired as single scans to better display subcellular features.

### BODIPY staining and oil body analysis

Meristematic tissue pieces were collected from 2–3-week-old plants and stained with BODIPY™ 493/503 (#11540326, Fisher Scientific). A 5 mM stock solution was prepared in DMSO and diluted to a 2 µM working solution in water. Tissues were incubated in this solution for 30 min in the dark, rinsed twice with water, and gently blotted dry on absorbent paper before mounting in perfluorodecalin, as described above for confocal imaging of plants aged 3–14 days. BODIPY fluorescence was detected using 488 nm excitation and a narrow emission window of 500–505 nm, alongside chlorophyll autofluorescence recorded under the settings described above.

Z-stacks were processed in Fiji. For OB size measurements, the BODIPY fluorescence channel was converted to grayscale, thresholded (values 0–20), and binarized with watershed separation. OBs were then quantified using the “Analyze Particles” function with a minimum size cutoff of 3 µm^2^. For fluorescence intensity measurements, regions of interest (ROIs) corresponding to OBs were generated from the binary image and transferred to the original grayscale image. The mean gray intensity and area of each ROI were then extracted using the “Set Measurements” and “Measure” functions. Data were compiled and plotted in PRISM software version 10 (GraphPad, La Jolla, California, USA).

### Extraction of terpenes and analysis by gas chromatography-mass spectrometry (GC-MS)

Approximately 200 mg of frozen tissue from 1-month-old *Marchantia polymorpha* plants was extracted with 1 mL of cold methanol containing 5 mM NaCl to quench enzymatic activity, following the method of Kumar et al.^26^. To enable quantification, 5 µg of dodecane (#297879, Sigma-Aldrich) was included as an internal standard. The tissue was disrupted in the solvent using a 3-mm stainless steel ball (#2205, Durston, High Wycombe, UK) and homogenized with a TissueLyser II (Qiagen). Samples were subsequently agitated on a Vibrax® shaker (#0002819002, IKA, Staufen, Germany) at 2000 rpm for 2 hours, and the resulting extracts were centrifuged to remove plant debris. The methanolic phase was then extracted once with hexane to isolate non-polar and medium-polar terpenes from the upper organic layer. Hexane extracts (200 µL) were analyzed on a Trace 1300 gas chromatograph (ThermoFisher) coupled to an ISQ 700 mass spectrometer (ThermoFisher) with a Zebron CD-5MS column (30 m × 0.25 mm × 0.25 μm; ThermoFisher). A splitless injection (1 µL) was conducted at 230 °C, and the GC-MS oven parameters were adapted from Kumar’s work^26^ as follows: the oven temperature was initially set to 70 °C and held for 3 min, followed by a ramp of 20 °C/min to 90 °C, a second ramp of 3 °C/min to 180 °C, a third ramp of 5 °C/min to 240 °C, and a final ramp of 20 °C/min to 300 °C, with a 6-min hold at this temperature. The MS began data acquisition after a 5.5-minute solvent delay, with the transfer line and ion source temperatures set at 250 °C and 270 °C, respectively. Scanning was conducted in full-scan mode (scan time: 0.17 s) over a mass range of 40–600 atomic mass units. Helium was used as the carrier gas, with a flow rate of 1.2 mL/min for sesquiterpenes in the taxadiene and β-amyrin datasets. For amorphadiene detection, the carrier flow rate was reduced to 0.9 mL/min. Chromatograms were processed using Chromeleon™ software (ThermoFisher), and terpene quantification was performed relative to the internal standard dodecane. Tentative identification of the major sesquiterpenes was achieved by comparing mass spectra to published data and by calculating retention indices using a single sample run with a C8–C40 alkane standard mixture (#40147-U, Superlco, Bellefonte, Pennsylvania, USA) as described by Adams^74^. Note that the endogenous terpene composition in CAM accessions may slightly differ from that reported for other accessions, such as Tak, in previous studies. The authentic standard of amorpha-4,11-diene was provided by Tomasz Czechowski and Ian Graham (University of York). To achieve more accurate quantification of β-amyrin and sterols, the extraction protocol and GC-MS method were modified. The same amount of frozen material was extracted with cold methanol supplemented with 10 µg of coprostan-3-ol (#C7578, Sigma-Aldrich) as an internal standard. The samples were extracted twice with hexane to obtain a total hexane volume of 1.5 mL, which was then dried under nitrogen flow in a Genevac™ concentrator EZ-2 (#EZ3P-23050-NN0, Genevac Ltd, Ipswich, UK). Extracts were derivatized with 50 µL 1-trimethylsilyl-imidazole (TMS; #92718, Superlco) and resuspended in 300 µL hexane. For GC-MS analysis, 200 µL of the derivatized samples was transferred into vials for direct injection. The method used for sterol/triterpene analysis was as follows: 130 °C hold for 2 min; ramp of 30 °C/min until 220 °C, then 2 °C/min until 300 °C, hold for 10 min. Solvent delay of 1 min before acquiring the MS data. The authentic standard of β-amyrin was provided by James Reed and Anne Osbourne (John Innes Center, Norwich, UK). For terpene quantification in Mp*abcg1*^*ge*^ and *Cas9*^*OE*^ lines, extractions were performed on genotyped plants from the second selection stage, as loss of the CRISPR genotype was observed during propagation from gemmae. Given this observation, we considered the possibility of chimerism^104,105^ and extracted as much biomass as was reasonably available from individual plants. This approach aimed to minimize variability, homogenize the material, and ensure accurate results. Box plots showing individual data points for each quantified terpene were created using PRISM software version 10 (GraphPad, La Jolla, California, USA), and the results of statistical comparisons were integrated into the visualizations. For lines expressing exogenous terpene synthases, statistical analyses were performed using one-way analysis of variance (ANOVA) to assess differences across gene combinations. Homogeneity of variances was evaluated using the Brown-Forsythe and Bartlett's tests, with Dunnett's multiple comparison test applied post-hoc to compare each gene combination to the respective control (_*Pro*_*MYB2:β-AS*, *TXS*, or *AMS*). The significance threshold (alpha) was set at 0.05. Four biological replicates expressing the constructs were analyzed to quantify both exogenous (β-amyrin and taxadiene) and endogenous compounds (*n* = 4), while eight biological replicates expressing the constructs were analyzed for amorphadiene lines, focusing on the quantification of endogenous terpenes since amorphadiene was not detected (*n* = 8). For comparisons of terpene levels between *abcg1* mutants (*n* = 6) and Cas9 controls (*n* = 5), Welch’s t test was used when variances were unequal, and unpaired *t* test was used when variances were equal, as determined by PRISM. Both tests accounted for differences in sample size between the two groups. Asterisks were added to the box plots to indicate statistically significant differences (*P* < 0.05) between Mp*abcg1*^*ge*^ mutants and *Cas9*^*OE*^ lines, as determined by Welch’s or unpaired *t* tests.

### Statistics and reproducibility

Statistical analyses were performed on data obtained from independent biological replicates, defined as independently generated transgenic lines or independently genotyped mutant plants. For experiments involving metabolite quantification, four to eight independent primary transformants were analyzed per construct, as indicated in the corresponding figure legends. For CRISPR-generated mutants, all available genotyped transformants were included. Statistical analyses were performed using GraphPad Prism. Where applicable, statistical significance was assessed using one-way ANOVA followed by Dunnett’s multiple comparisons test, unpaired two-tailed Student’s *t* tests, or Welch’s *t* tests, as specified in the figure legends. Quantitative data are presented as box plots showing individual data points.

For imaging experiments lacking formal statistical analysis, transformations were performed in independent rounds and multiple plants were analyzed per construct to confirm reproducibility of localization patterns.

### Reporting summary

Further information on research design is available in the Nature Portfolio Reporting Summary linked to this article.

## Supplementary information

Supplementary information

Description of Additional Supplementary Files

Supplementary data 1 to 5

Supplementary data 6

Reporting Summary

## Data Availability

Source data for terpene quantification and oil body measurements using BODIPY staining are provided as Supplementary Data 1–5. Sequences and vectors used to generate the constructs for plant transformation are provided in Supplementary Data 6. Original Leica.lif files containing confocal microscopy images are available from the corresponding authors upon reasonable request.
