# Supplementary material for: Spatial distribution of isoprenoid enzymes and MpABCG1 transporter influences sesquiterpene accumulation in Marchantia polymorpha oil bodies
Source: Commun Biol. 2026 Mar 2;9:521. doi: 10.1038/s42003-025-09508-4 (PMC13068942; doi:10.1038/s42003-025-09508-4)
Supplement: Supplementary file 1 — Supplementary information [file 42003_2025_9508_MOESM1_ESM.pdf]

Spatial distribution of isoprenoid enzymes and MpABCG1 transporter influence sesquiterpene accumulation in *Marchantia polymorpha* oil bodies.

Edith C. F. Forestier, Paola Asprilla, Ignacy Bonter, Facundo Romani, Eftychios Frangedakis and Jim Haseloff.

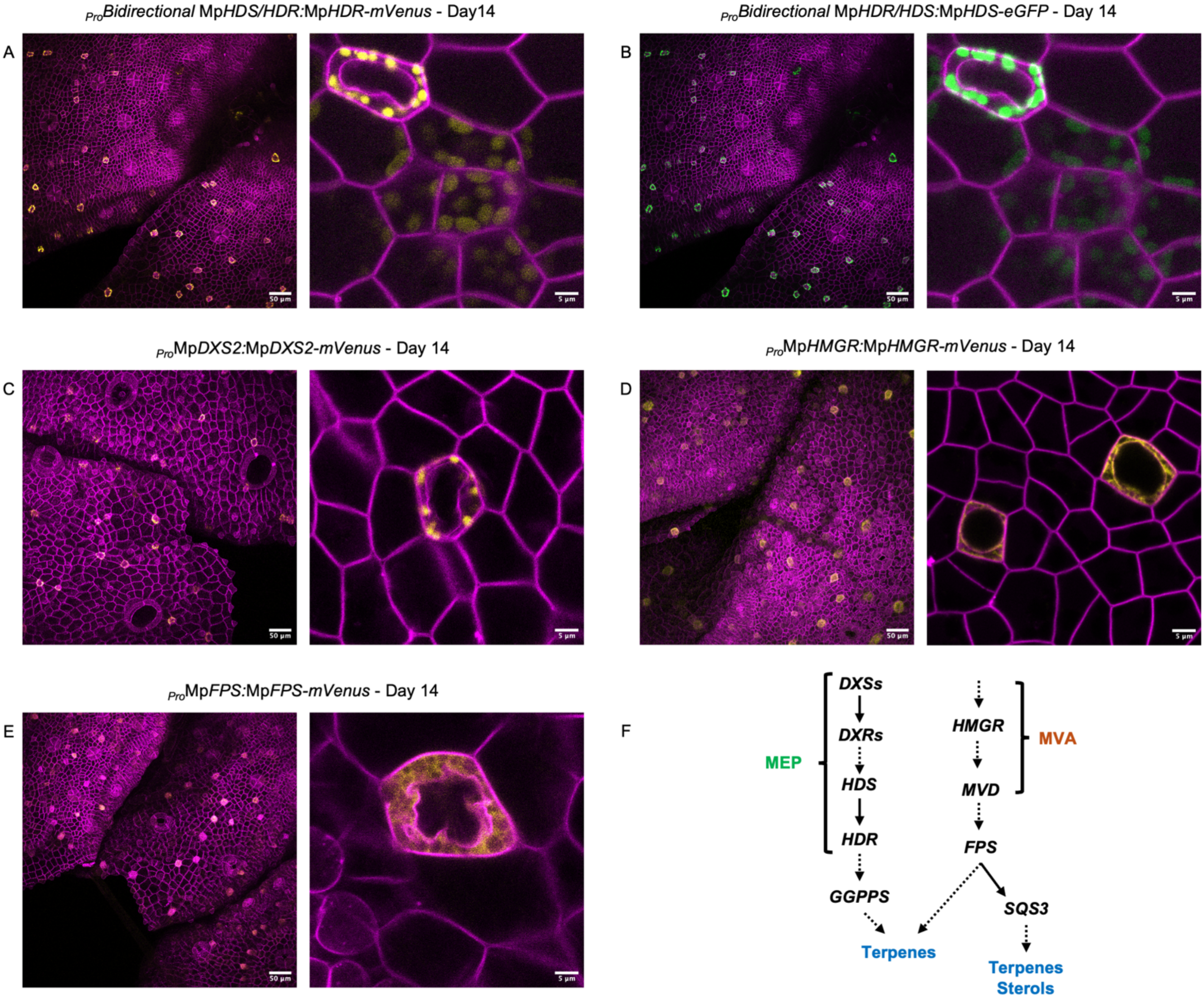

Figure S1. Confocal imaging of translational reporters for selected *Marchantia polymorpha* isoprenoid biosynthetic genes in the meristem area of 14-day-old plants. (A) $_{Pro}$*Bidirectional* Mp*HDS/HDR:HDR-mVenus*, (B) $_{Pro}$*Bidirectional* Mp*HDR/HDS:HDS-eGFP*, (C) $_{Pro}$Mp*DXS2:DXS2-mVenus*, (D) $_{Pro}$Mp*HMGR:HMGR-mVenus* and (E) $_{Pro}$Mp*FPS:FPS-mVenus*. The mVenus (yellow) or eGFP (green) signals indicate the subcellular localization of the respective enzymes, while mScarlet fluorescence (purple) delineates cellular boundaries. Each construct is represented by two panels: the left panels (scale bar: 50 μm) show a broader view of the meristem area, while the right panels (scale bar: 5 μm) provide a zoomed-in view of subcellular localization. (F) Simplified biosynthetic pathway highlighting the enzymatic steps studied using translational and transcriptional reporters studied in this work.

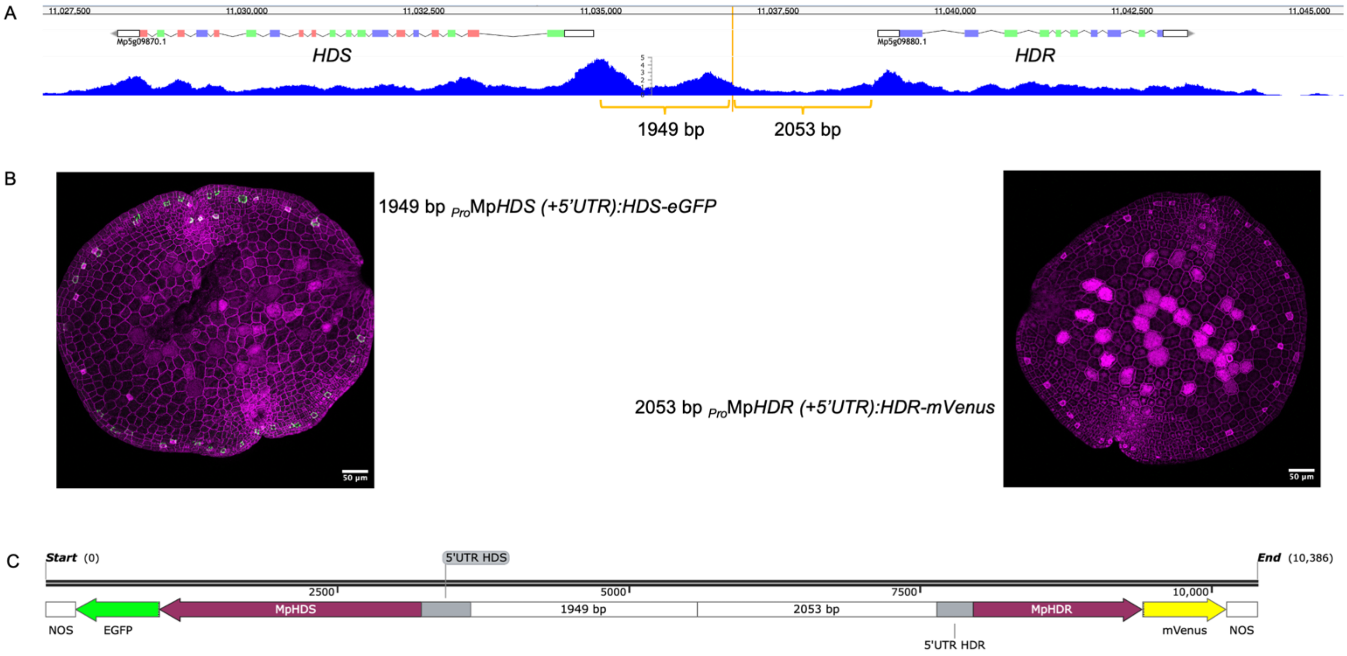

Figure S2. Bidirectionality of the Mp*HDS-HDR* promoter region and design of the bidirectional translational reporter.
(A) Gene structure of Mp*HDS* and Mp*HDR*, showing the region between the two genes with ATAC-seq peaks (blue) extracted from the Marchantia.info database (Tak accession, version 6.1). The orange line indicates a boundary splitting the intergenic region to create separate promoters for Mp*HDS* (left) and Mp*HDR* (right). (B) Confocal imaging of translational reporters $_{Pro}$Mp*HDS:HDS-eGFP* (left) and $_{Pro}$Mp*HDR:HDR-mVenus* (right) in day 0 gemmae. The mScarlet fluorescence (purple) delineates cell boundaries, while eGFP (green) indicates subcellular localization of MpHDS. MpHDR-mVenus fluorescence is not detected. Scale bar: 50 µm. (C) Schematic representation of the bidirectional translational reporter construct designed to drive expression of Mp*HDS* and Mp*HDR,* with mVenus (yellow) and eGFP (green) reporters fused to Mp*HDR* and Mp*HDS*, respectively. The construct design was performed using SnapGene software.

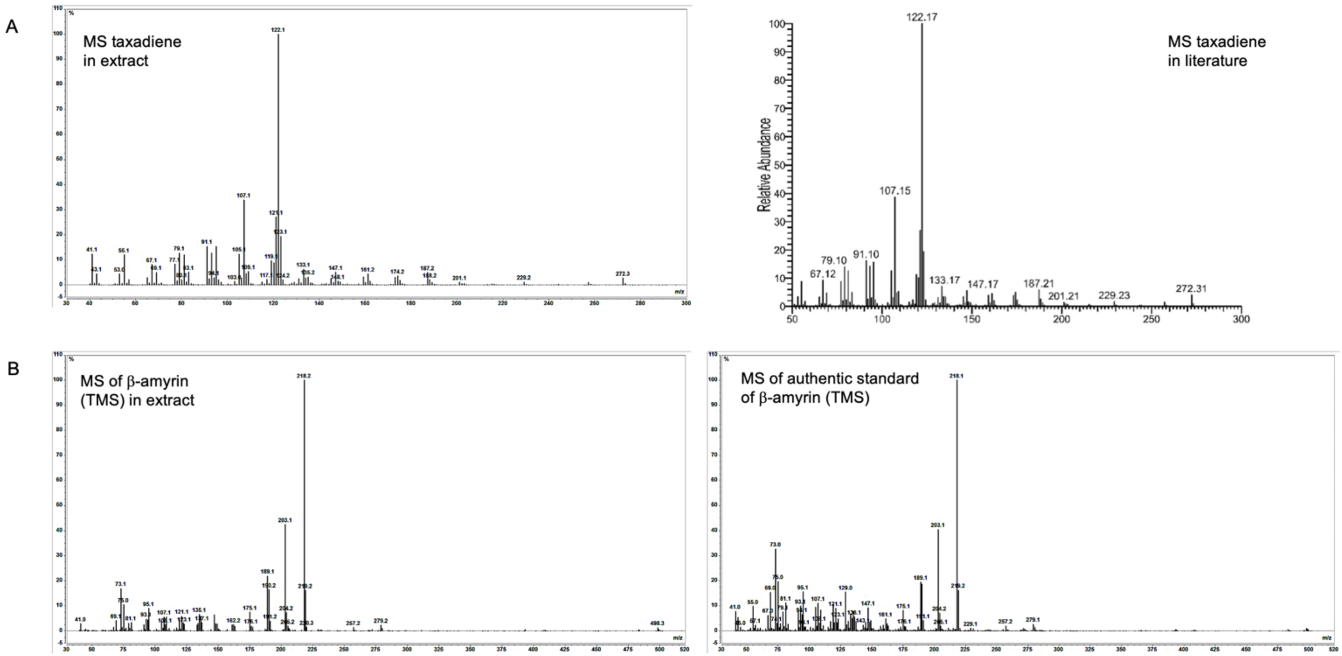

Figure S3. Mass spectra of taxadiene and trimethylsilyl-derivatized β-amyrin identified in *Marchantia polymorpha* extracts.

(A) Mass spectrum of taxadiene detected in *M. polymorpha* terpene extracts (left). The molecular ion (M^+) at 272 and fragment ion at 122 are characteristic of taxadiene. As no commercial standard is available for taxadiene, the reference spectrum (right) is taken from Nowrouzi et al[1]. for comparison. (B) Mass spectrum of trimethylsilyl-derivatized β-amyrin detected in *M. polymorpha* terpene extracts (left) compared to an authentic β-amyrin standard (right).

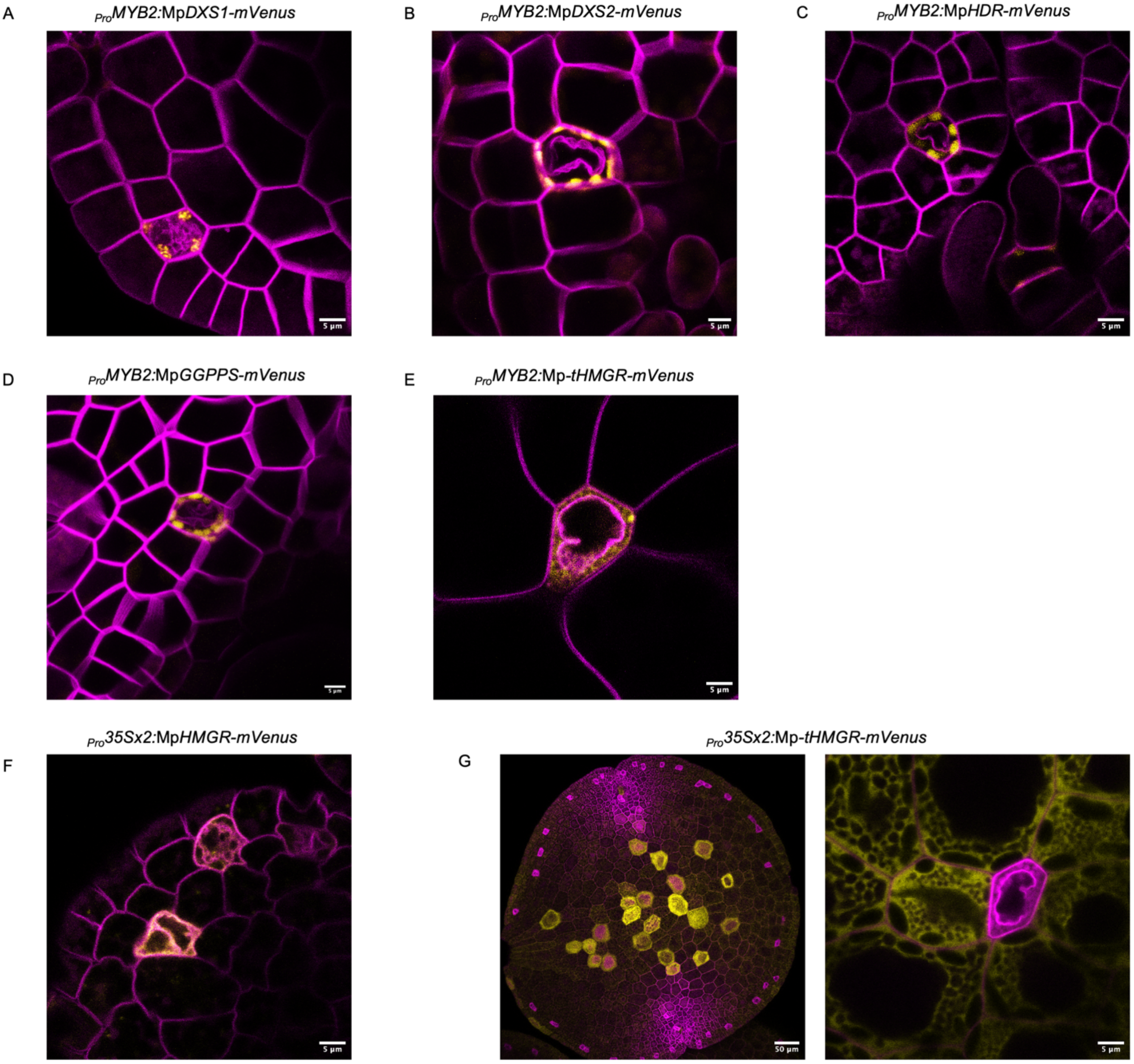

Figure S4. Subcellular localization of precursor enzymes expressed under alternative promoters.

Subcellular localization of *Marchantia* mVenus-tagged fusion proteins driven by the oil body-specific $_{Pro}$*MYB2* promoter for (A) MpDXS1, (B) MpDXS2, (C) MpHDR, (D) MpGGPPS, and (E) Mp-tHMGR (scale bars: 5 µm). Subcellular localization of MpHMGR (F) and Mp-tHMGR (G) fusion proteins expressed under the *2x35S* promoter. For $_{Pro}$*2x35S:*Mp-*tHMGR-mVenus* (G), a broader view of gemmae is shown (left panel; scale bar: 50 µm), along with a higher-magnification image (right panel; scale bar: 5 µm). Cell boundaries are marked by mScarlet fluorescence (purple).

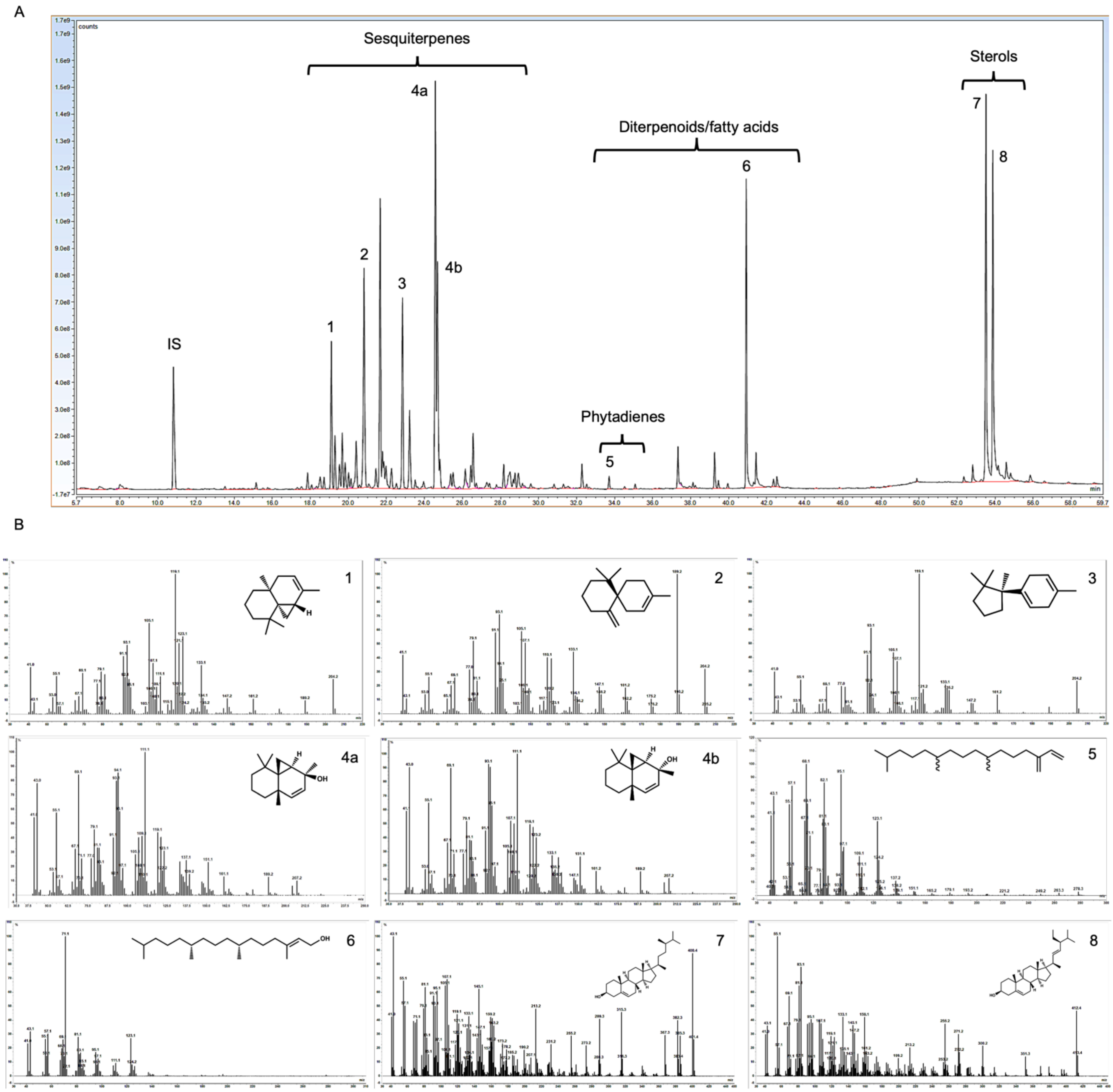

Figure S5. Total ion chromatogram (TIC) and mass spectra of terpenes and phytosterols detected in *Marchantia polymorpha* extracts.

(A) TIC of a methanol extract followed by hexane extraction from a 2-month-old, non-axenic *Marchantia polymorpha* culture. The selected peaks were tentatively identified as cis-thujopsene (1), β-chamigrene (2), γ-cuprenene (3), thujopsan-2α-ol (4a), thujopsan-2β-ol (4b), neophytadiene (5), phytol (6), campesterol (7), and stigmasterol (8). IS denotes the internal standard used for quantification. (B) Mass spectra of the labeled peaks in (A) with corresponding chemical structures of the putative compounds. Sesquiterpenes (1–4b) and diterpenes/fatty acids (5–6) were tentatively identified using Kovats retention indices and mass spectral data as detailed in Table S3[2,3], while phytosterols (7 and 8) were identified based on their characteristic mass spectra.

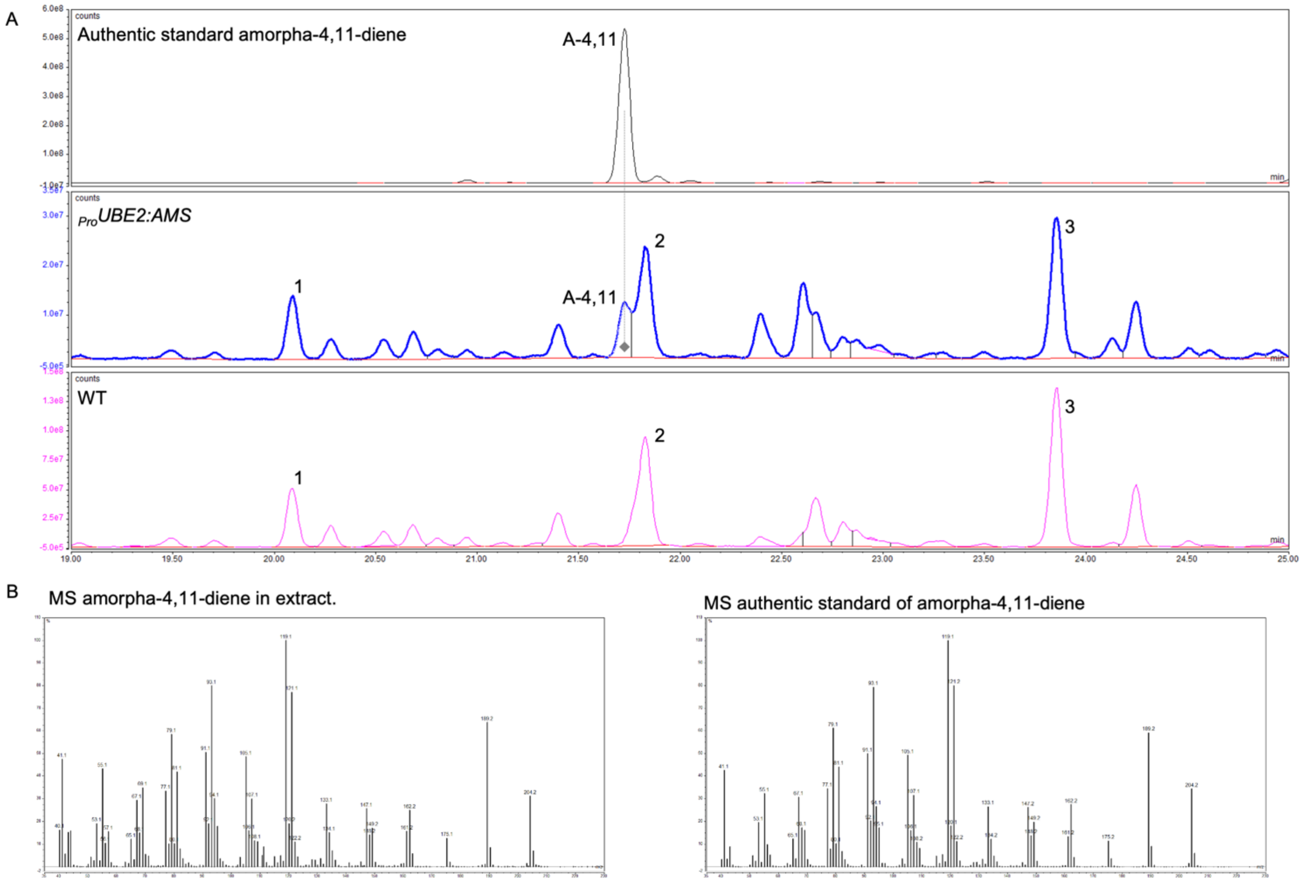

Figure S6. Identification of amorpha-4,11-diene in *Marchantia polymorpha* expressing *Artemisia annua* AMS under $_{Pro}$*UBE2*.

(A) TICs of terpene extracts from WT *Marchantia polymorpha* (lower chromatogram), plants expressing $_{Pro}$*UBE2:AMS* (middle chromatogram), and the authentic standard of amorpha-4,11-diene (upper chromatogram). A new peak labeled A-4,11 appears in the middle chromatogram, matching the retention time of the authentic standard. Peaks labeled 1, 2, and 3 correspond to the endogenous sesquiterpenes cis-thujopsene, β-chamigrene, and γ-cuprenene, respectively. (B) Mass spectra of amorpha-4,11-diene in the extract (left) and the authentic standard (right), demonstrating matching fragmentation patterns.

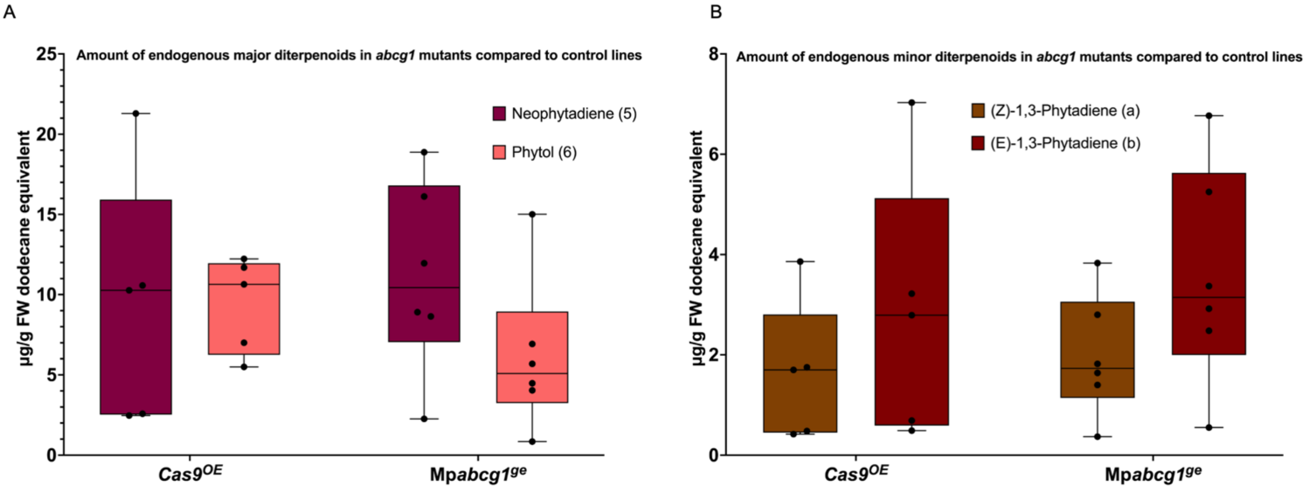

Figure S7. Quantification of phytol and its derivatives in Mp*abcg1*^{ge} mutant and *Cas9*^{OE} control lines.

(A) Levels of major fatty acids/diterpenoids neophytadiene (5) and phytol (6), in *Cas9*^{OE} (n=5) and Mp*abcg1*^{ge} lines (n=6). (B) Quantification of the minor fatty acids/diterpenoids (*Z*)-1,3-phytadiene (a) and (*E*)-1,3-phytadiene (b) in *Cas9*^{OE} controls (n=5) and Mp*abcg1*^{ge} mutant lines (n=6). Box plots display individual data points for each compound, with bars representing the median and interquartile range.

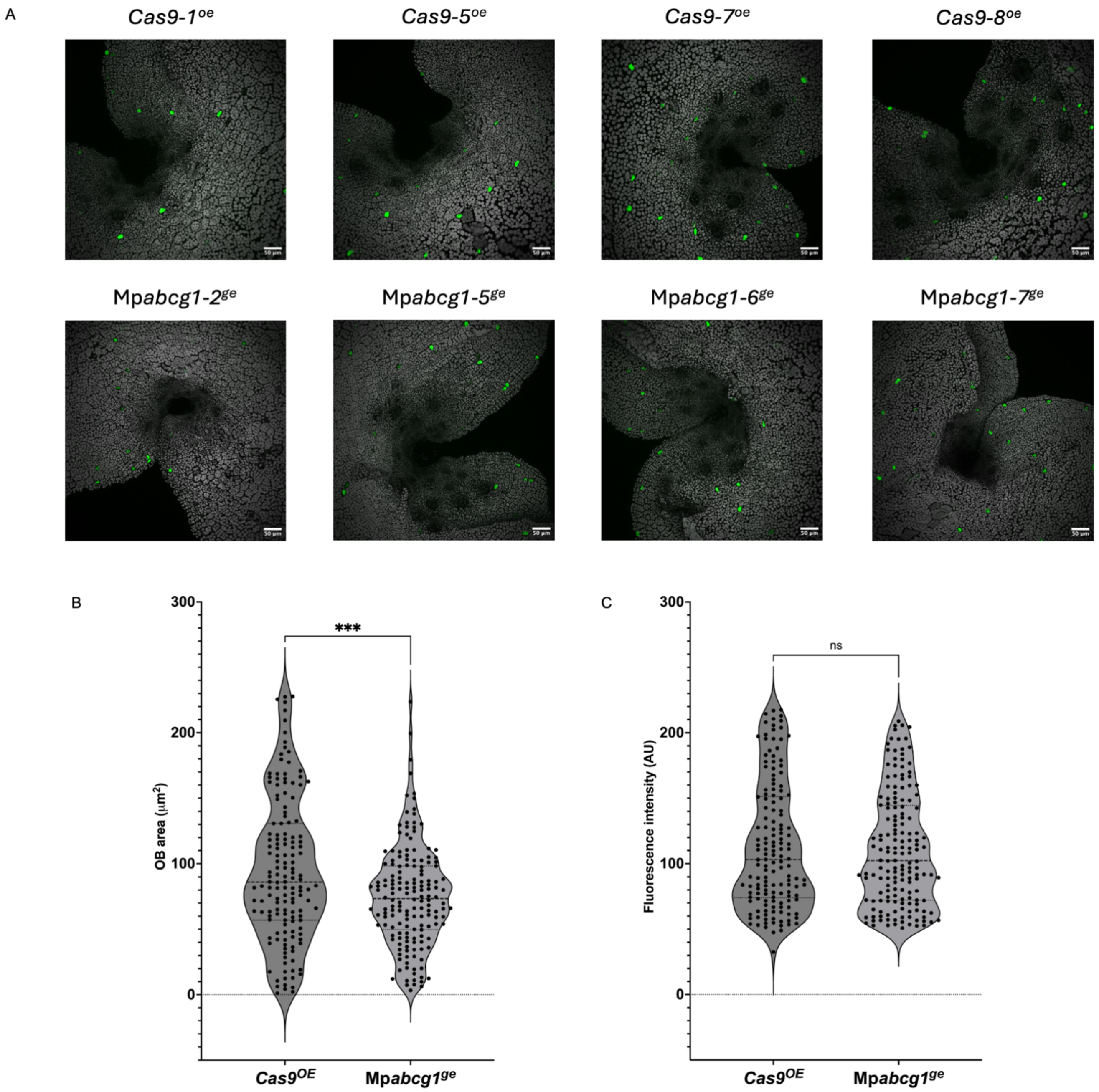

Figure S8. Effect of Mp*ABCG1* disruption on oil bodies in *Marchantia polymorpha*.
(A) Representative confocal images of two to three pieces of young meristematic tissues s coming from four independent *Marchantia polymorpha* Cas9 overexpression control lines (*Cas9^{OE}*) and Mp*abcg1* CRISPR knockout lines (Mp*abcg1^{ge}*), following Bodipy staining to visualize oil bodies (OBs). Scale bars: 100 µm. (B) Quantification of OB area in *Cas9^{OE}* (n = 150 OBs) and Mp*abcg1^{ge}* (n = 164 OBs) lines. (C) Quantification of Bodipy fluorescence intensity per OB in the same samples as (B). Statistical comparisons were performed using Welch's tests (**p < 0.001, ns = not significant).

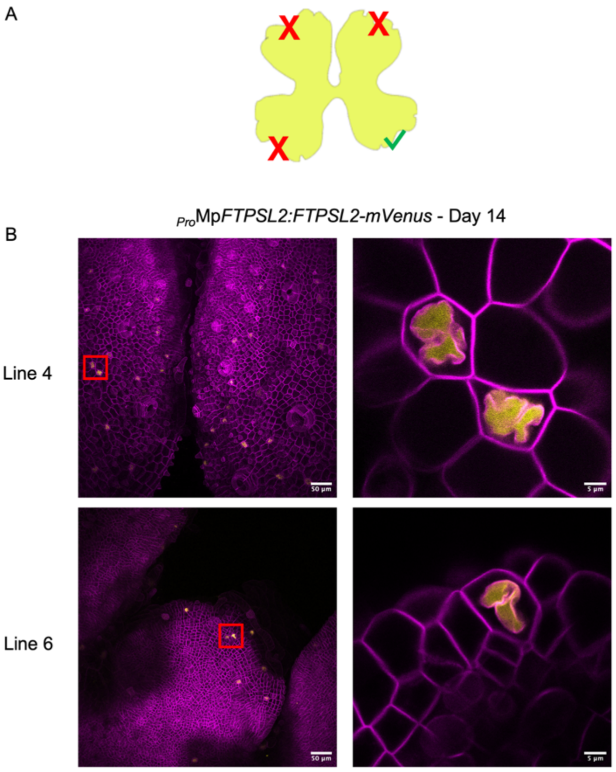

Figure S9. Localization of the endogenous sesquiterpene synthase MpFTPSL2 driven by its own promoter.
(A). Illustration of a 14-day-old *Marchantia* thallus, adapted from Marchantia.info, under a Creative Commons Attribution (CC-BY 4.0) licence, showing that the mVenus signal was detected in only one of the four thallus lobes (green checkmark) and absent in the others (red X). (B) Confocal imaging of the translational reporter $_{Pro}$Mp*FTPSL2:FTPSL2-mVenus* in two independent 14-day-old plants (lines 4 and 6). Left panels show the meristematic region (scale bar: 50 µm), with red squares indicating the areas magnified in the right panels (scale bar: 5 µm).

A

Guide	Strand	Position	DNA sequence
1	+	17	GATGGAAAGCTTGCCCTCCG
2	+	18	ATGGAAAGCTTGCCCTCCGT
3	+	19	TGGAAAGCTTGCCCTCCGTG
4	+	245	AGAACTCAAAGACATGAGTA
5	+	293	GCTATTTCAGATGAACCCAG
6	+	296	ATTTCAGATGAACCCAGAGG

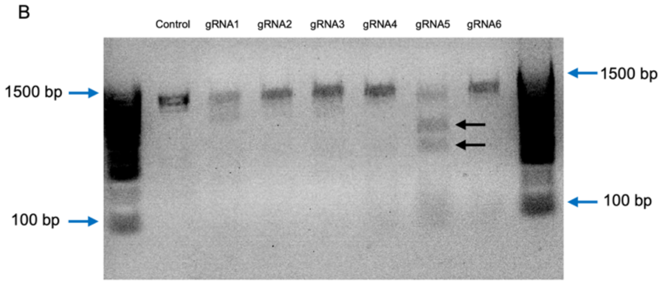

Figure S10. In vitro Cas9 cleavage assay to select guide RNAs targeting Mp*ABCG1*.
(A) Candidate gRNA sequences targeting the first 300 bp of the Mp*ABCG1* coding sequence. The table lists the strand orientation and target position for each gRNA. The sequence targeted by gRNA5 is highlighted in yellow as it showed the highest cleavage efficiency in panel B. (B) Agarose gel showing in vitro cleavage products of PCR-amplified Mp*ABCG1* DNA incubated with recombinant Cas9 and each of the six gRNAs. Control = PCR product with no gRNA. Cleaved fragments are indicated by black arrows for gRNA5. Blue arrows indicate the positions of 1500 bp (upper) and 100 bp (lower) DNA fragments. The gel shown corresponds to the complete gel image as acquired using the gel documentation system: no additional cropping or image manipulation was performed after image acquisition.

Supplementary table 1. Accession number of genes described in this study with their known or putative function.

Abbreviation	Accession number (marchantia.info)	Putative function
Mp*DXS1*	Mp2g09130	MEP pathway genes
Mp*DXS2*	Mp4g14720	
Mp*DXR1*	Mp6g04420	
Mp*DXR2*	Mp8g01240	
Mp*MCT*	Mp3g06780	
Mp*CMK*	Mp1g16290	
Mp*MDS*	Mp1g29200	
Mp*HDS*	Mp5g09870	
Mp*HDR*	Mp5g09880	
Mp*IDI1*	Mp4g00610	
Mp*IDS2* /Mp*GGPPS*	Mp2g13280	Diterpene scaffold formation.
Mp*IDS3*	Mp5g21200	Very low expressed GGPPS.
Mp*IDS4*	Mp5g20580	Large polyprenol scaffold formation such as ubiquinone.
Mp*IDS5*	Mp1g06110	Monoterpene scaffold formation. Phosphate moiety in *trans* .
Mp*ACT2*	Mp5g23030	MVA pathway genes
Mp*HMGS*	Mp4g22640	
Mp*HMG* /Mp*HMGR*	Mp2g07980	
Mp*MK*	Mp4g13410	
Mp*PMK*	Mp8g14400	
Mp*MVD*	Mp3g01340	
Mp*IDI2*	Mp6g12890	
Mp*IDS1* /Mp*FPS*	Mp3g22530	Sesquiterpene scaffold formation.
Mp*SQS3*	Mp4g01910	Triterpene/sterol scaffold formation.
MpSQS1	Mp2g21100	Carotenoid scaffold formation.
Mp*SQS2*	Mp3g14160	Unknown role in terpenoid synthesis.

Supplementary Table 2. Promoter and 5′UTR lengths of *Marchantia polymorpha* genes used in

<table>
<tr><th>Gene studied</th><th>Promoter length (bp)</th><th>5'UTR length (bp)</th><th>Total length (bp)</th></tr>
<tr><td>MpDXS1</td><td>882</td><td>476</td><td>1358</td></tr>
<tr><td>MpDXS2</td><td>685</td><td>351</td><td>1036</td></tr>
<tr><td>MpDXR1</td><td>1383</td><td>396</td><td>1779</td></tr>
<tr><td>MpDXR2</td><td>1551</td><td>1213</td><td>2764</td></tr>
<tr><td>MpHDS</td><td>1949</td><td>420</td><td rowspan="2">4735</td></tr>
<tr><td>MpHDR</td><td>2053</td><td>313</td></tr>
<tr><td>MpGGPPS</td><td>490</td><td>477</td><td>967</td></tr>
<tr><td>MpHMGR</td><td>2107</td><td>669</td><td>2776</td></tr>
<tr><td>MpMVD</td><td>696</td><td>542</td><td>1238</td></tr>
<tr><td>MpFPS</td><td>805</td><td>148</td><td>953</td></tr>
<tr><td>MpSQS3</td><td>2344</td><td>628</td><td>2972</td></tr>
<tr><td>MpFTPSL1</td><td>1220</td><td>222</td><td>1442</td></tr>
<tr><td>MpFTPSL2</td><td>2215</td><td>41</td><td>2256</td></tr>
</table>

Supplementary Table 3. Identification of major terpenes detected by GC-MS in *Marchantia polymorpha* extracts based on retention indices and mass spectra.

	RT (min)	Calculated RI	Adams RI	Identity	MS reference*
1	19.10	1435	1429	cis-Thujopsene	https://webbook.nist.gov/cgi/cbook.cgi?ID=C470406&Mask=200#Mass-Spec
2	20.82	1480	1476	β-Chamigrene	https://webbook.nist.gov/cgi/cbook.cgi?ID=C18431828&Mask=200
3	22.85	1535	1532	γ-Cuprenene	Adams, 2007
4a	24.57	1581	1586	Thujopsan-2α-ol**	https://spectrabase.com/. John Wiley & Sons, Inc.
4b	24.68	1583	1588	Thujopsan-2β-ol**	n/a
5	33.71	1841	1841	Neophytadiene	Santos *et al*., 2017
6	40.92	2113	***2111	Phytol	https://webbook.nist.gov/cgi/cbook.cgi?ID=C150867&Mask=200

* Reference spectra used for comparison with spectra in Figure S5
** Tentative assignment to the stereoisomers thujopsan-2-α-ol and thujopsan-2-β-ol based on close elution, mutually near identical MS, and Kovats RI close to literature
*** RI from NIST database

RT = retention time
RI = retention index

Supplementary table 4. Primers used in this study.

Part amplified	Acceptor vector	Forward primer (5'-3')	Reverse primer (5'-3')
MpHMGR CDS12	L0	AAAGCTCTTCGTCTCTAATGAAGTCGGGAGATGGACTCAAGACTTATG	AAAGCTCTTCGTCTCTCGAAGCCTCAACTTTGGGGGAT
Mp-tHMGR CDS12	L0	AAAGCTCTTCGTCTCTAATGTCCCTGAAGGAGCATGAGA	Same than above
Mp-tHMGR CDS	L1	TTTGGTCTCTAATGTCCCTGAAGGAGCATGAGAAT	TTTGGTCTCTAAGCCTACTCAACTTTGGGGGATTT
Pp-tHMGR CDS	L1	AAAGGTCTCTAATGTGTGACAATGAGGATGAGGACATT	TTTGGTCTCTAAGCTCAGGCGGAAGTAGTCTTAGTTG
MpFPS CDS	L0	AAAGCTCTTCGTCTCTAATGCTATGGTTACAGAGGGGATTTGC	AAAGCTCTTCGTCTCTAAGCTCATTTTTGTCGCTTGTAAATCTTTCC
MpFPS CDS12	L0	Same than above	AAAGCTCTTCGTCTCTCGAAGCTTTTTGTCGCTTGTAAATCTTT
AMS CDS12	L1	AAAGGTCTCTAATGAGCCTGACCGAGGAGAAG	AAAGGTCTCTCGAAGCGATGCTCATAGGGTA
BAS CDS12	L0	AAAGCTCTTCGTCTCTAATGTGGAGACTGAAGATCGG	AAAGCTCTTCGTCTCTCGAAGCGGTGCTGTT
MpDXS1 CDS12	L0	AAAGCTCTTCGTCTCTAATGGCGACTAGTGTTGTGAATTCCA	AAAGCTCTTCGTCTCTCGAAGCCGACATCAACTGAAG
MpDXS2 CDS12	L0	AAAGCTCTTCGTCTCTAATGGCGGCCAGCATCG	AAAGCTCTTCGTCTCTCGAAGCCTTTGATAATGTCAAAGTTTCA
MpHDS CDS12	L0	AAAGCTCTTCGTCTCTAATGCAGACCATGAATGTTACAG	AAAGCTCTTCGTCTCTCGAAGCTGCTTCAACTACC
MpHDR CDS12	L0	AAAGCTCTTCGTCTCTAATGGCTACCACCACGACTGCT	AAAGCTCTTCGTCTCTCGAAGCAGCTGGAACCTGA
MpGGPPS CDS12	L0	AAAGCTCTTCGTCTCTAATGGGAAGCGGGGTTGCAG	AAAGCTCTTCGTCTCTCGAAGCATTTTGCCTACTAGCGATGTAGT
TXS CDS12	L0	AAAGCTCTTCGTCTCTAATGGCTCAGCTGAGCTTCAACG	AAAGCTCTTCGTCTCTCGAAGCCACTTGGATAGGATCG
Pro MpHDR:HDR-eGFP-NOS cassette	L1	AAAGGTCTCTCTTATATAGCGAAGTAAACAGCGGACAC	AAAGGTCTCTAGCGTCGATCTAGTAACATAGATGACACC
Pro MpHDS:HDS-mVenus-NOS cassette	L1	AAAGGTCTCTGGAGTCGATCTAGTAACATAGATGACACCG	AAAGGTCTCTTAAGAAACATGTCCACTTTTGTATCGGAATTC
Mp*abcg1* single guide RNA	L2	TCGGCTATTTCAGATGAACCCAGGT	AAAACCTGGGTTCATCTGAAATAGC
Mp*abcg1*^{ge} genotyping	NA	AAGACTCCGGATCCGAGGG	CCTCAACTTTGGGCAGGTCA
Cas9^{OE} genotyping	NA	CTCGCTCTCGCTCACATGAT	GGTAGGTTCCGAGAGATGCG

References.

1. Nowrouzi, B. *et al.* Enhanced production of taxadiene in Saccharomyces cerevisiae. *Microbial Cell Factories* **19**, 200 (2020).
2. Adams, R. P. *Identification of Essential Oil Components by Gas Chromatography Mass Spectroscopy*. (Allured Publishing Corporation, Carol Stream, Ill, 2007).
3. Santos, S. A. O. *et al.* Lipophilic Fraction of Cultivated Bifurcaria bifurcata R. Ross: Detailed Composition and In Vitro Prospection of Current Challenging Bioactive Properties. *Marine Drugs* **15**, 340 (2017).
