# Supplementary material for: Spatial distribution of isoprenoid enzymes and MpABCG1 transporter influences sesquiterpene accumulation in Marchantia polymorpha oil bodies
Source: Commun Biol. 2026 Mar 2;9:521. doi: 10.1038/s42003-025-09508-4 (PMC13068942; doi:10.1038/s42003-025-09508-4)
Supplement: Supplementary file 2 — Description of Additional Supplementary Files [file 42003_2025_9508_MOESM2_ESM.pdf]

Description of Additional Supplementary Files:

File name: Supplementary Data 1

Description: Numerical source data underlying graphs presented in Figure 3D and 3E.

File name: Supplementary Data 2

Description: Numerical source data underlying graphs and charts presented in Figure 3D and 3E.

File name: Supplementary Data 3

Description: Numerical source data underlying graphs and charts presented in Figure 4A and 4C.

File name: Supplementary Data 4

Description: Numerical source data underlying graphs and charts presented in Figure 5C and S7.

File name: Supplementary Data 5

Description: Numerical source data underlying graphs and charts presented in Figure S8

File name: Supplementary Data 6

Description: Sequence information and design syntax for all genetic constructs generated in this study, provided as a multi-tab Excel file.
