# Supplementary material for: Spatial distribution of isoprenoid enzymes and MpABCG1 transporter influences sesquiterpene accumulation in Marchantia polymorpha oil bodies
Source: Commun Biol. 2026 Mar 2;9:521. doi: 10.1038/s42003-025-09508-4 (PMC13068942; doi:10.1038/s42003-025-09508-4)
Supplement: Supplementary file 5 — Reporting Summary [file 42003_2025_9508_MOESM5_ESM.pdf]

nature portfolio

Corresponding author(s): Jim Haseloff

Last updated by author(s): 2025/11/11

Reporting Summary

Nature Portfolio wishes to improve the reproducibility of the work that we publish. This form provides structure for consistency and transparency in reporting. For further information on Nature Portfolio policies, see our Editorial Policies and the Editorial Policy Checklist.

Statistics

For all statistical analyses, confirm that the following items are present in the figure legend, table legend, main text, or Methods section.

n/a	Confirmed	
☐	☒	The exact sample size (*n*) for each experimental group/condition, given as a discrete number and unit of measurement
☐	☒	A statement on whether measurements were taken from distinct samples or whether the same sample was measured repeatedly
☐	☒	The statistical test(s) used AND whether they are one- or two-sided<br>*Only common tests should be described solely by name; describe more complex techniques in the Methods section.*
☒	☐	A description of all covariates tested
☐	☒	A description of any assumptions or corrections, such as tests of normality and adjustment for multiple comparisons
☐	☒	A full description of the statistical parameters including central tendency (e.g. means) or other basic estimates (e.g. regression coefficient) AND variation (e.g. standard deviation) or associated estimates of uncertainty (e.g. confidence intervals)
☒	☐	For null hypothesis testing, the test statistic (e.g. *F*, *t*, *r*) with confidence intervals, effect sizes, degrees of freedom and *P* value noted<br>*Give P values as exact values whenever suitable.*
☒	☐	For Bayesian analysis, information on the choice of priors and Markov chain Monte Carlo settings
☒	☐	For hierarchical and complex designs, identification of the appropriate level for tests and full reporting of outcomes
☒	☐	Estimates of effect sizes (e.g. Cohen's *d*, Pearson's *r*), indicating how they were calculated

Our web collection on statistics for biologists contains articles on many of the points above.

Software and code

Policy information about availability of computer code

Data collection	Confocal microscopy images were acquired using Leica LAS X software (Leica Microsystems, version unspecified). Gas chromatography–mass spectrometry data were collected with ThermoFisher Chromeleon™ software (version 7).
Data analysis	Quantitative data were analyzed using GraphPad Prism version 10 (GraphPad Software, La Jolla, CA) and Microsoft Excel (Office 365) for basic data handling. Image analysis was performed with Fiji/ImageJ version 2.14. Bioinformatic analyses were conducted using NCBI BLAST, DeepLoc 2.1, and UniProt online tools.

For manuscripts utilizing custom algorithms or software that are central to the research but not yet described in published literature, software must be made available to editors and reviewers. We strongly encourage code deposition in a community repository (e.g. GitHub). See the Nature Portfolio guidelines for submitting code & software for further information.

Data

Policy information about availability of data

All manuscripts must include a data availability statement. This statement should provide the following information, where applicable:

- Accession codes, unique identifiers, or web links for publicly available datasets
- A description of any restrictions on data availability
- For clinical datasets or third party data, please ensure that the statement adheres to our policy

All data supporting the findings of this study are provided within the article and its Supplementary Datasets. Raw quantitative data for metabolite analyses and

vector sequences (including cloning syntax and transcription unit arrangements) are available in the Supplementary Information. Raw confocal microscopy image files are available from the corresponding authors upon reasonable request due to file size limitations. All protein and DNA sequences from Marchantia polymorpha used in this study are publicly available through the Marchantia.info / MarpolBase database.

Research involving human participants, their data, or biological material

Policy information about studies with human participants or human data. See also policy information about sex, gender (identity/presentation), and sexual orientation and race, ethnicity and racism.

Reporting on sex and gender	n/a
Reporting on race, ethnicity, or other socially relevant groupings	n/a
Population characteristics	n/a
Recruitment	n/a
Ethics oversight	n/a

Note that full information on the approval of the study protocol must also be provided in the manuscript.

Field-specific reporting

Please select the one below that is the best fit for your research. If you are not sure, read the appropriate sections before making your selection.

☒ Life sciences ☐ Behavioural & social sciences ☐ Ecological, evolutionary & environmental sciences

For a reference copy of the document with all sections, see nature.com/documents/nr-reporting-summary-flat.pdf

Life sciences study design

All studies must disclose on these points even when the disclosure is negative.

Sample size	No formal statistical power calculation was performed. For metabolite quantification, eight independent primary transformants were initially screened for compound presence, and four representative lines containing the compound of interest were selected for quantitative analysis. This number ensured that all analyzed plants expressed the construct and could be grown simultaneously under homogeneous conditions while maintaining sufficient spacing on the plate.<br>For constructs that did not yield detectable amorphadiene, all eight transformants were analyzed to evaluate the effect of co-expressed genes on endogenous terpene levels. For CRISPR mutants, all available genotyped transformants were analyzed. These sample sizes were sufficient to capture biological variation between independent lines and to perform statistical comparisons using ANOVA or t-tests.
Data exclusions	No data were excluded from the analyses. All measurements from the selected biological replicates were included in the statistical analyses. The choice of lines for metabolite quantification (as described under "Sample size") was made prior to data collection and therefore does not constitute post hoc exclusion.
Replication	Independent biological replication was ensured through the use of multiple independent transformants per construct. For metabolite quantification, four to eight independent primary transformants were analyzed per construct, providing reproducible trends across lines. For confocal imaging, transformations were performed twice independently, and at least four plants were imaged per construct in each round, yielding consistent localization patterns. All key experimental findings were successfully reproduced. The only exception was the ProFTPSL2:FTPSL2-mVenus reporter, which showed a restricted localization pattern in two lines; this observation was included in the Supplementary Information and identified as requiring further confirmation.
Randomization	For metabolite quantification, eight independent transformants were randomly selected from the initial transformation plate without bias regarding plant size or morphology. For lines expressing fluorescent reporters, transformants were pre-screened under a stereomicroscope to confirm fluorescence before analysis, but selection was otherwise random with respect to growth characteristics. No additional group allocation was required, as all constructs were analyzed independently under identical growth and sampling conditions.
Blinding	Blinding was not relevant to this study, as data collection relied on objective analytical methods that are not influenced by investigator expectation.

Reporting for specific materials, systems and methods

We require information from authors about some types of materials, experimental systems and methods used in many studies. Here, indicate whether each material, system or method listed is relevant to your study. If you are not sure if a list item applies to your research, read the appropriate section before selecting a response.

Materials & experimental systems

n/a	Involved in the study
☒	☐ Antibodies
☒	☐ Eukaryotic cell lines
☒	☐ Palaeontology and archaeology
☒	☐ Animals and other organisms
☒	☐ Clinical data
☒	☐ Dual use research of concern
☐	☒ Plants

Methods

n/a	Involved in the study
☒	☐ ChIP-seq
☒	☐ Flow cytometry
☒	☐ MRI-based neuroimaging

Plants

Seed stocks	The study used Marchantia polymorpha accessions Cam1 (male) and Cam2 (female), maintained in the Haseloff Lab (University of Cambridge) and widely used in previous publications from this group. These accessions originated from the grounds of the Department of Plant Sciences, University of Cambridge. Plants were propagated vegetatively via gemmae or tissue cuttings. Spores used for genetic transformation were obtained through manual fertilization between Cam1 and Cam2. No formal stock centre or catalogue number applies.
Novel plant genotypes	Novel Marchantia polymorpha genotypes were generated by Agrobacterium tumefaciens–mediated transformation of spores following established protocols for the Cam1/Cam2 accessions. Binary vectors were assembled using the Loop cloning system and introduced into Agrobacterium strain GV3101. Transformants were selected on hygromycin-containing Gamborg B5 medium, and typically eight independent primary transformants were obtained per construct. Four representative lines were propagated clonally from gemmae and used for metabolite quantification and subcellular localization. CRISPR–Cas9 gene-edited lines targeting MpABCG1 were generated using the Sauret-Gueto et al. single-guide acceptor vector system carrying Streptococcus pyogenes Cas9. Guide RNA#5 (5'-GTTCTTCGAGGGTGATGAGG-3') targeted the first coding exon, and editing introduced indels resulting in premature stop codons. Independent genotyped primary transformants were analyzed alongside Cas9-only control lines for metabolite and phenotypic assays.
Authentication	All binary plasmids were fully sequence-verified at Plasmidsaurus prior to transformation. Transgenic lines were authenticated by confirming construct-specific phenotypes: fluorescence signal for reporter constructs and metabolite production for lines expressing exogenous terpene synthases. Consistent expression or metabolite profiles were observed across multiple independent transformants, confirming reproducibility and minimizing the likelihood of secondary insertion effects. For MpABCG1 CRISPR–Cas9 mutants, editing at the target locus was verified by Sanger sequencing, and all independent alleles displayed comparable reductions in sesquiterpene levels. Although chimerism could not be completely excluded, as described in the manuscript, analyses were performed on the total genotyped tissue to minimize its impact, and the consistent phenotypes observed across multiple independent mutant lines indicate that the effects result from disruption of MpABCG1 rather than off-target edits.
